# Casbane Diterpenes from Red Sea Coral *Sinularia polydactyla*

**DOI:** 10.3390/molecules21030308

**Published:** 2016-03-03

**Authors:** Mohamed-Elamir F. Hegazy, Tarik A. Mohamed, Abdelsamed I. Elshamy, Montaser A. Al-Hammady, Shinji Ohta, Paul W. Paré

**Affiliations:** 1 Department of Phytochemistry, National Research Centre, El-Tahrir Street, Dokki, Giza 12622, Egypt; elamir77@live.com (M.-E.F.H.); tarik.nrc83@yahoo.com (T.A.M.); 2 Department of Natural Compound Chemistry, National Research Centre, El-Tahrir Street, Dokki, Giza 12622, Egypt; elshamynrc@yahoo.com; 3 National Institute of Oceanography and Fisheries, Red Sea Branch, Hurghada 84511, Egypt; coralreef_niof1@yahoo.com; 4 Graduate School of Biosphere Science, Hiroshima University, 1-7-1 Kagamiyama, Higashi-Hiroshima 739-8521, Japan; ohta@hiroshima-u.ac.jp; 5 Department of Chemistry and Biochemistry, Texas Tech University, Lubbock, TX 79409, USA

**Keywords:** soft coral, alcyoniidae, *Sinularia polydactyla*, diterpenes

## Abstract

The soft coral genus *Sinularia* is a rich source of bioactive metabolites containing a diverse array of chemical structures. A solvent extract of *Sinularia polydactyla* resulted in the isolation of three new casbane diterpenes: sinularcasbane M (**1**), sinularcasbane N (**2**) and sinularcasbane O (**3**); in addition, known metabolites (**4**–**5**) were isolated. Compounds were elucidated on the basis of spectroscopic analyses; the absolute configuration was confirmed by X-ray analysis.

## 1. Introduction

**Figure 1:**
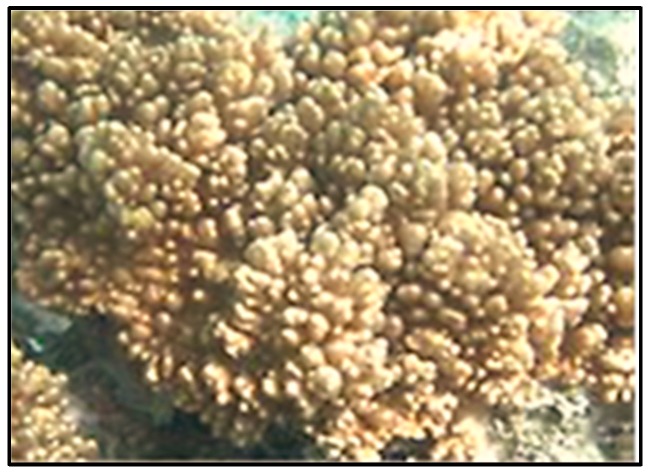
Soft coral *Sinularia polydactyla*.

**Figure 2:**
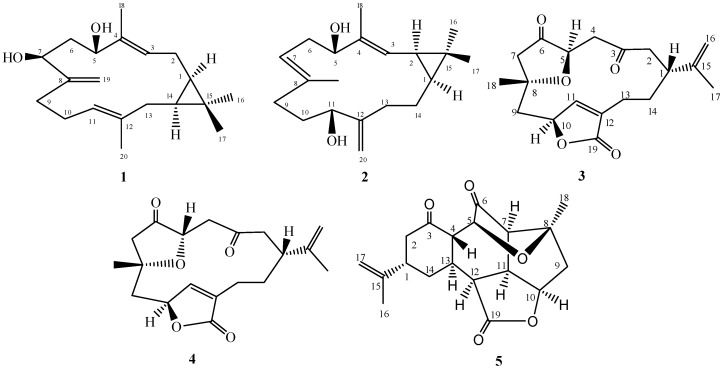
Structures of metabolites **1**–**5**.

In Alcyonacean soft coral, the genus *Sinularia* is a rich source of diverse natural products with over 500 metabolites including sesquiterpenes, diterpenes, polyhydroxylated steroids, alkaloids and polyamines already having been chemically characterized [1,2,3,4,5]. *Sinularia* consists of almost 90 species of which approximately 50 have been profiled for biological activity; such studies have established that the genus is a rich source of secondary metabolites with biological properties including cytotoxic, anti-inflammatory and antimicrobial activities [5,6,7,8,9,10]. While *Sinularia* species occur in marine waters around the world, *S. polydactylais* (Figure 1) is endemic to the Red Sea. Although the marine environment contains extensive reef formations, this ecosystem is not as well characterized in terms of chemical studies of marine organisms compared to other large coral reef systems [11]. As a part of a comprehensive chemical inventory of marine natural products from Red Sea soft coral [12,13,14], herein, we reported the chemical characterization of solvent-extracted casbane diterpenes from *S. polydactyla* (Figure 2). Casbane-type diterpene structures are related to the 14-membered cembrane ring system except for the dimethyl-cyclopropyl moiety instead of an isopropyl residue fused to the ring. These extremely-rare natural products are predominantly isolated from select coral species [15]. Several soft coral casbanes have been isolated from *Sinularia depressa* with biologically active 10-hydroxydepressin exhibiting cytotoxicity against several tumor cell lines with IC_50_ values near 50 μM [16].

## 2. Results and Discussion

**Figure 3:**
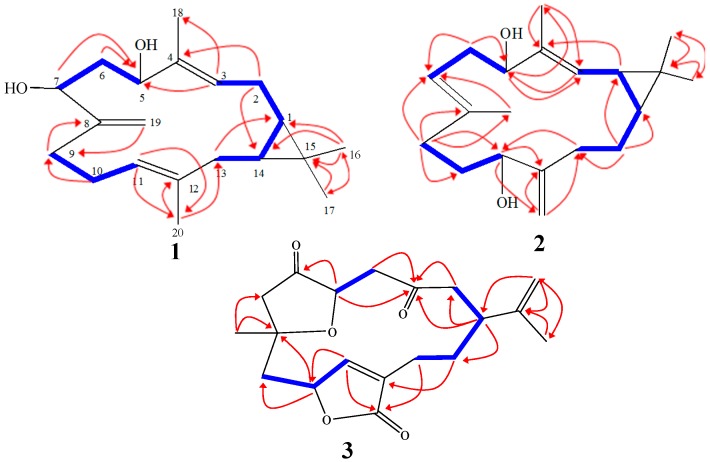
Selected ^1^H-^1^H COSY (—) and HMBC (→) correlations of **1**–**3**.

Compound **1** was obtained as a colorless oil with a negative optical rotation (${\left[\alpha \right]}_{D}^{25}$ − 9.6 in MeOH). HR-ESI-FTMS analysis exhibited a molecular ion peak at *m*/*z* 327.2293 [M + Na]^+^ corresponding to a molecular formula of C_20_H_32_O_2_ (calcd. for C_20_H_32_O_2_Na, 327.2300) with five degrees of unsaturation. The IR spectrum exhibited bands for hydroxyl and olefinic substituents at 3409, and 1650 cm^−l^, respectively. The ^1^H-NMR spectrum showed four methyl signals, including two olefinic methyls at δ_H_ (1.56 brs and 1.74 brs, each 3H) and two tertiary methyls at δ_H_ (1.00 s and 1.06 s, each 3H). Olefinic protons at δ_H_ 5.02 (brd, *J* = 8.3 Hz) and 5.19 (brd, *J* = 8.3 Hz) were attributed to two trisubstituted double bonds; exomethylene singlets were observed at δ_H_ 4.89 and 5.04. In addition, two secondary oxygenated proton signals appeared at δ_H_ 3.93 (brd, *J* = 11.1 Hz) and 4.39 (dd, *J* = 10.0, 4.3 Hz). The ^13^C-NMR spectrum displayed 20 carbon resonances which were classified by DEPT spectra as four methyls, six methylenes, six methines and four quaternary carbons. In addition, two oxygenated carbons at δ_C_ 71.2 and 77.0 and six olefinic carbons at δ_C_ 125.4, 137.9, 124.4, 136.9, 154.0, and 109.8 were observed. A casbane-type diterpene skeleton was predicted based on NMR data comparisons of analogous structures previously isolated from the same genus [4,15,16]. Characteristic signals at δ_C_ 25.0, 31.5, and 20.1 were proposed to constitute a cyclopropane moiety assigned to C-1, C-14 and C-15, respectively by DQF-COSY and HMBC analyses. The DQF-COSY showed correlations between signals at δ_H_ 0.69 (td, *J* = 10.9, 2.6 Hz) and 0.67 (td, *J* = 10.9, 4.0 Hz) with methylene mutiplet signals δ_H_ 1.26 and 1.80/2.19, respectively. Additionally, HMBC data confirmed these carbon assignments with correlations with signals at δ_H_ 0.69 (td, *J* = 10.9 and 2.6 Hz, H-1), 0.67 (td, *J* = 10.9 and 4.0 Hz, H-14), 1.26 (m, H_2_-2) and 1.80/2.19 (m, H_2_-13). Two singlet signals at δ_C_ 15.8 and 28.7 were assigned to methyl groups at C-16 and C-17 respectively and attached to C-15 based on HMBC correlations (Figure 3). The olefinic signal at δ_H_ 5.19 (d, *J* = 8.3 Hz) was assigned to H-3 based on an observed DQF-COSY correlation with H-2 and HMBC correlation between H-3 and an olefinic methyl and oxygenated methine at δ_C_ 10.5 (C-18) and 77.0 (C-5), respectively. Additionally, HMBC correlations allowed for the assignment of H-3 (δ_H_ 5.19), H_3_-18 (δ_H_ 1.74), and H-5 (δ_H_ 4.39), (Figure 3). DQF-COSY correlations with H-5 allowed for a methylene signal assignment at δ_H_ 1.60 (ddd, *J* = 13.8, 10.0, 2.2 Hz, H-6)/1.94 (ddd, *J* = 13.8, 10.0, 2.2 Hz, H-6) as well as the oxygenated signal at δ_H_ 3.93 (brd, *J* = 11.1 Hz, H-7). C-7 (δ_C_ 71.2) exhibited a HMBC correlation with exocyclic broad singlets at δ_H_ 4.89 (H-19) and 5.04 (H-19). HMBC correlations were observed between C-19 and δ_H_ 2.07 (m, H-9)/2.19 (m, H-9) as well as between H-9 and δ_C_ 154 (C-8). DQF-COSY correlation between H-9 and δ_H_ 2.07/2.19 (m) and 5.02 (brd, *J* = 8.3 Hz) allowed for the assignments of H-10 and H-11, respectively and the endocyclic proton H-11 coupled with δ_C_ 40.7 (C-13) in the HMBC spectrum. Finally, HMBC correlations were observed between H-11 and δ_C_ 15.8 (C-20) as well as H-20 and endocyclic signal δ_C_ 136.0 (C-12).

**Figure 4:**
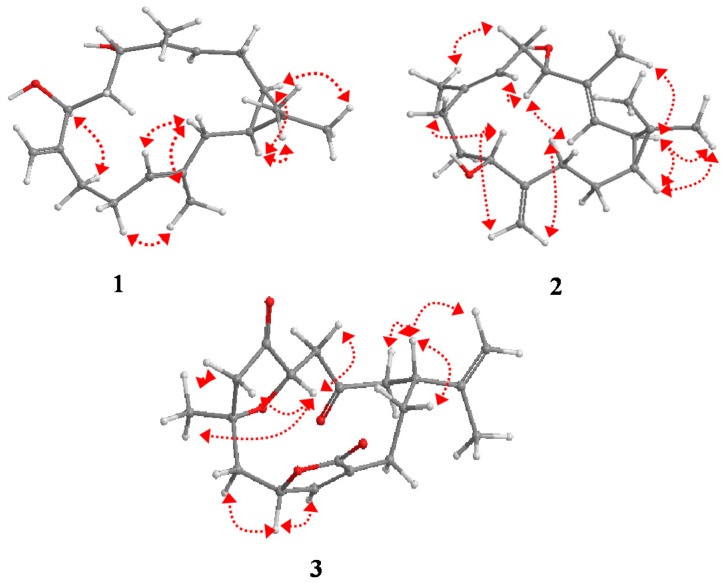
Energy-minimized 3D structure and NOESY correlations (→) for **1**–**3**.

NOESY correlations observed between H-1, H-14 and H-16, indicated protons oriented on the same side consistent with a *cis* ring junction. The spectrum showed two proton signals attributed to 1,2-cyclopropane moiety at δ_H_ 0.69 (td, *J* = 10.9, 2.6 Hz, H-1) and 0.67 (td, *J* = 10.9, 4.0 Hz, H-14). Due to the flexible nature of the 14-membered macrocyclic ring, the relative configuration of the hydroxyl groups at C-5 and C-7 could not be determined by NOESY; however coupling constants for H-5/H-6a and H-6b/H-7 of 4.3 and 11.1 Hz respectively, indicated an *cis*-configuration for the hydroxyls. H-1α, H-3 and H-5 NOE correlations led to an assignment of H-5α and H-7α (Figure 4) which was confirmed by chemical shifts of δ_C_ 15.6 and 28.7 for C-16 and C-17 methyl groups respectively, and consistent with previously reported *cis*-fused casbane derivatives [4,15]. Based on this spectral analysis, **1** was identified as 5β,7β-dihydroxy-1α,14α-casba-3,8(19),11-triene, named here as a sinularcasbane M.

**Figure 5:**
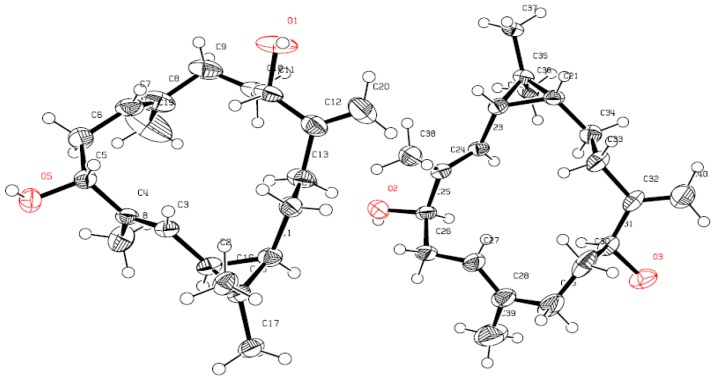
ORTEP depictions of **2** with oxygens (O1–O5) labeled in red.

Compound **2** was obtained as colorless crystals with a negative optical rotation (${\left[\alpha \right]}_{D}^{25}$ − 15.5 in MeOH). HR-ESI-FTMS analysis showed a molecular ion peak at *m*/*z* 327.2295 [M + Na]^+^ corresponding to a molecular formula of C_20_H_32_O_2_ (calcd. for C_20_H_32_O_2_Na, 327.2300) with five degrees of unsaturation. The IR spectrum exhibited bands for hydroxyl and olefinic groups at 3409, and 1650 cm^−l^, respectively. Spectroscopic data was similar to **1** except for oxygenated and cyclopropane proton signals. The DQF-COSY spectrum showed a correlation between one of the cyclopropane signal δ_H_ 1.28 (dd, *J* = 10.1, 8.9 Hz) and the olefinic signal at δ_H_ 5.09 (dd, *J* = 10.1, 1.2 Hz) introducing the possibility of the cyclopropane ring or double bond shifting. The second cyclopropane signal at δ_H_ 0.77 (ddd, *J* = 11.9, 8.9, 2.7 Hz) correlates with the methylene protons at δ_H_ 1.13 (1H, dddd, *J* = 13.4, 12.8, 11.9, 3.4 Hz) and 1.55 (1H, dddd, *J* = 14.0, 13.4, 4.9, 2.7 Hz) which is consistent with the cyclopropane moiety being shifted to C-1 and C-2 and the olefinic bond remaining at C-3 and C-4. Also consistent with this shift, the olefinic proton H-3 correlates with an oxygenated signal at δ_C_ 79.5 in the HMBC spectrum allowing for a hydroxyl group to be located at C-5 (δ_H_ 3.97, dd, *J* = 10.7, 4.6 Hz). H-5 correlated with the methylene signals at δ_H_ 2.31 (ddd, *J* = 13.4, 10.7, 7.9 Hz)/2.38 (brdd, *J* = 13.4, 4.6 Hz) in DQF-COSY, allowing for the assignment of H-6. H-6 showed a correlation with the olefinic methine signal at δ_H_ 4.86 (brdd, *J* = 7.9, 1.2 Hz, H-7) as well as the broad methyl singlet at δ_H_ 1.63 (H_3_-19) in DQF-COSY and HMBC, respectively. Similar spectra for **1** and **2** allowed for the exomethylene to be assigned to C-12/C-20. HMBC correlation between C-12 and δ_H_ 3.90 (dd, 7.6, 5.2 Hz, H-11) as well DQF-COSY correlations between H-11 and δ_H_ 1.52/1.66 (m, H-10) and H-10 with δ_H_ 1.98 (dd, 15.6, 7.6, H-9)/2.09 (ddd, 15.6, 13.1, 3.1, H-9) established methylene groups at C-9-C-10 and a hydroxyl at C-11. A HMBC correlation between C-20 and δ_H_ 1.66 (m, H-13)/2.14 (ddd, 12.8, 12.8, 4.9, H-13) along with DQF-COSY correlations between H-13 and δ_H_ 1.13 (dddd, 13.4, 12.8, 11.9, 3.4, H-14)/1.55 (dddd, 14.0, 13.4, 4.9, 2.7, H-14), δ_H_ 0.77 (ddd, 11.9, 8.9, 2.7, H-1) and δ_H_ 1.28 (dd, 10.1, 8.9, H-2) allowed for the assignment of the other NMR signals (Figure 3). The relative configuration of hydroxyl group at C-11 was based on biogenetic considerations of casbane derivatives published by Yin, *et al.* in 2013, Yang, *et al.*, 2015, and Li, *et al.* in 2010 [4,15,16], which was supported by coupling constant and NOESY experiments to be in the β position. Consequently, the relative configurations of H-1 and H-2 showed interaction with H-16 in NOESY experiments to be in the α position (Figure 3). Absolute configuration was established by X-ray crystallography (Figure 5). Therefore, **2** was assigned as 5*S*,11*R*-dihydroxy-1*S*,2*R*-casba-3,7,12(20)-triene, a new natural product named here as sinularcasbane N.

Compound **3** was obtained as a colorless oil with a positive optical rotation (${\left[\alpha \right]}_{D}^{25}$ + 10.2 in MeOH). HR-ESI-FTMS analysis showed a molecular ion peak at *m*/*z* 355.1516 [M + Na]^+^ (calcd. for C_19_H_24_O_5_Na: 355.1521), consistent with a molecular formula C_19_H_24_O_5_, indicating eight degrees of unsaturation. Spectroscopic data was similar to previously reported **4** by Saitman, *et al.* in 2011, except around C-5, raising the possibility that **3** was an epimer of **4**. Spectral shift differences included a downfield shift of H-5 (δ_H_ 4.42 d, *J* = 10.3 Hz) and an upfield shift of H-4 (2.61 and 2.49); downfield shifts for C-5, C-7, and C-4 in comparison with **4** were also observed [17,18,19]. The relative configuration of the four chiral centers at C-1, C-5, C-8, and C-10 were also determined by ^13^C signal comparisons as well as observed NOESY correlations (Figure 4). Assignment of H-1 to a β-orientation was based on Alcyonacea biogenesis of cembrane-type compounds [5,19]. In the NOESY spectrum, H-1 correlates with H-14β (δ_H_ 1.75 m) and one proton of C-2 methylene (δ_H_ 2.24, H-2β). NOESY correlations between H-5 (δ_H_ 4.42, d) with H-11α at (δ_H_ 7.20, s), H-4α at (δ_H_ 2.57, m), and H-10α at (δ_H_ 5.15 s) confirmed an α-orientation for H-5, indicating that epimerization occurs at C-5. Therefore, the norcembranoid was assigned to be the epimer of scabrolide F (sinularcasbane O).

In addition, previously reported metabolites scabrolide F (**4**) [18] and ineleganolide (**5**) [20] were identified by NMR and MS comparisons with literature values.

## 3. Materials and Methods

### 3.1. General Information

Specific rotation was measured with a Horiba SEPA-300 digital polarimeter (Kyoto, Japan, *l* = 5 cm) and IR spectra were collected on a Shimadzu FTIR-8100 spectrometer (Kyoto, Japan). For X-ray, a Bruker SMART-APEX II ULTRA (Billerica, MA, USA) was used. ESI-MS and HR-ESI-MS were carried out using a Thermo Fisher Scientific LTQ Orbitrap XL mass spectrometer (Waltham, MA, USA), and ^1^H (600 MHz) and ^13^C (150 MHz) NMR spectra were recorded on a JEOL JNM-ECA 600 spectrometer (Tokyo, Japan) with tetramethylsilane as an internal standard. Purification was run on a Shimadzu HPLC system equipped with a RID-10A refractive index detector and compound separation was performed on YMC-Pack ODS-A (Tokyo, Japan, 250 × 4.6 mm i.d.) and (250 × 20 mm i.d.) columns for analytical and preparative separation, respectively. Chromatography separation included normal-phase silica BW-200 (Fuji Silysia Chemical, Ltd., Kasugai, Japan, 150–350 mesh) and ODS reverse phase Chromatorex DM1020T (Fuji Silysia Chemical, Ltd., 100–200 mesh) columns as well as silica gel 60_F254_ (Merck, Darmstadt, Germany, 0.25 mm) and RP-18 WF_254S_ (Merck, 0.25 mm) TLC plates with spots developed with heating of H_2_SO_4_-MeOH (1:9) sprayed plates.

### 3.2. Animal Material

Soft coral *S. polydactyla* was collected from the Egyptian Red Sea off the coast of Hurghada in March 2013. The soft coral was identified by Montaser A. Al-Hammady with a voucher specimen (03RS100) deposited in the National Institute of Oceanography and Fisheries, marine biological station, Hurghada, Egypt.

### 3.3. Extraction and Separation

Frozen soft coral (6.5 kg, total wet weight) was chopped into small pieces and extracted with methylene chloride/methanol (1:1) at room temperature (4 L × 5 times). The combined extracts were concentrated *in vacuo* to a brown gum. The dried material (243 g) was subjected to gravity chromatography in a silica gel column (6 cm × 120 cm) eluting with *n*-hexane (3000 mL) followed by a gradient of *n*-hexane-CH_2_Cl_2_ up to 100% CH_2_Cl_2_ and CH_2_Cl_2_–MeOH up to 50% MeOH (3000 mL each of the solvent mixture). The *n*-hexane/CH_2_Cl_2_ (1:2) fraction (2.2 g) eluted with *n*-hexane/EtOAc (6:1) was subjected to silica gel column separation. Fractions were obtained and combined into two main sub-fractions, A and B, according to a TLC profile. Sub-fraction A was re-purified by reversed-phase HPLC using MeOH/H_2_O (70%:30%) to afford **3** (11 mg), **4** (18 mg) and **5** (22 mg).

Sub-fraction B was re-purified by reversed-phase HPLC (Shimadzu HPLC system equipped with a RID-10A refractive index detector and compound separation was performed on YMC-Pack ODS-A (250 × 10 mm i.d.) column for separation) using MeOH/H_2_O (65%:35%) to afford **6** (10 mg). Sub-fraction C was re-purified by reversed-phase HPLC using MeOH/H_2_O (60%:30%) to afford **1** (11 mg) and **2** (18 mg).

*Sinularcasbane M* (**1**): colorless oil; ${\left[\alpha \right]}_{D}^{25}$ = −9.6 (*c* 0.01, CHCl_3_); ^1^H-NMR and ^13^C-NMR data, see Table 1; HR-ESI-FTMS [M + Na]^+^ *m*/*z* 327.2293 (calc. 327.2300, C_20_H_32_O_2_Na); also see Figure S1.

*Sinularcasbane N* (**2**): colorless crystal; ${\left[\alpha \right]}_{D}^{25}$ = −15.5 (*c* 0.01, CHCl_3_); ^1^H-NMR and ^13^C-NMR data, see Table 1; HR-ESI-FTMS [M + Na]^+^ *m*/*z* 327.2295 (calc. 327.2300, C_20_H_32_O_2_Na); also see Figure S1.

#### X-ray Crystallography Data

Single crystal X-ray analysis established the complete structure and absolute configuration of **2** and the crystal data are summarized as follows: C_40_H_61_O_3_, formula wt. 589.88, triclinic, space group, a = 9.437(4) Å, b = 9.499(4) Å, c = 10.543(4) Å, α = 98.763(6)°, β = 95.390(5)°, γ = 99.422(5)°, volume = 914.6(6) Å, are based upon the refinement of the XYZ-centroids of 5460 reflections above 20 σ(I) with 5.389° < 2θ < 41.63°. Data were corrected for absorption effects using the multi-scan method (SADABS). The ratio of minimum to maximum apparent transmission was 0.750. D_cacld_ = 1.071 g/cm^3^, crystal size 0.369 mm^3^ × 0.344 mm^3^ × 0.267 mm^3^. A total of 720 frames were collected, exposure time was 0.40 h and the frames were integrated with the Bruker SAINT Software package (V8.34A; Bruker AXS Inc., Madison, Wisconsin, USA, 2013) using a narrow-frame algorithm. The integration of the data using a triclinic unit cell yielded a total of 5460 reflections to a maximum θ angle of 27.85° (0.76 Å resolution), of which 4717 were independent (average redundancy 1.158, completeness = 95.8%, R_int_ = 2.69%, R_sig_ = 6.64%) and 3571 (75.70%) were greater than 2σ (F2). CCDC 1407158 contains the supplementary crystallographic data for this paper. These data can be obtained free of charge via http://www.ccdc.cam.ac.uk/conts/retrieving.html (or from the CCDC, 12 Union Road, Cambridge CB2 1EZ, UK; Fax: +44 1223 336033; E-mail: deposit@ccdc.cam.ac.uk.).

*Sinularcasbane O* (**3**): colorless oil; ${\left[\alpha \right]}_{D}^{25}$ = +10.2 (*c* 0.01, CHCl_3_); ^1^H-NMR and ^13^C-NMR data, see Table 1; HR-ESI-FTMS [M + Na]^+^ *m*/*z* 355.1517 (calc. 355.1521 C_19_H_24_O_5_Na); also see Figure S1.

*Scabrolide F* (**4**): ${\left[\alpha \right]}_{D}^{25}$ = −12.3 (*c* 0.01, CHCl_3_), HR-ESI-FTMS [M + Na]^+^ *m*/*z* 355.1516 (calc. 355.1521 C_19_H_24_O_5_Na).

*Ineleganolide* (**5**): colorless oil; ${\left[\alpha \right]}_{D}^{25}$ = −35.0 (*c* 0.01, CHCl_3_), HR-ESI-FTMS [M + Na]^+^ *m*/*z* 353.1370 (calc. 353.1365 C_19_H_22_O_5_Na).

## 4. Conclusions

The methylene chloride/methanol (1:1) extract from the Red Sea coral *S. polydactyla* afforded three new metabolites, sinularcasbane M (**1**), sinularcasbane N (**2**) and sinularcasbane O (**3**). The structures were elucidated by spectroscopic analyses; the absolute configuration of **2** was confirmed by X-ray analysis.

## Figures and Tables

**Table 1:** ^1^H- and ^13^C-NMR spectral data recorded in CDCl_3_, at 600 and 150 MHz, respectively.

Position	1		2		3		4
	δ_H_ (*J* in Hz)	δ_C_	δ_H_ (*J* in Hz)	δ_C_	δ_H_ (*J* in Hz)	δ_C_	δ_C_
1	0.69 (td, 10.9, 2.6)	25.0	0.77 (ddd, 11.9, 8.9, 2.7)	31.1	2.58 m	40.8	38.8
2	1.26 (m)	29.6	1.28 (dd, 10.1, 8.9)	25.2	2.94 d (3.42), 2.96 d (2.04)	50.2	50.2
3	5.19 (brd, 8.3)	125.4	5.09 (dd, 10.1, 1.2)	125.6		208.4	208.4
4		137.9		136.8	2.47 m, 2.57 m	44.0	44.0
5	4.39 ( dd, 10.0, 4.3)	77.0	3.97 (dd, 10.7, 4.6)	79.5	4.12 d (3.42), 4.14 (2.76)	74.8	77.8
6	1.60 (ddd, 13.8, 11.1, 4.3)	40.2	2.31 (ddd, 13.4, 10.7, 7.9)	33.2		212.7	212.5
	1.94 (ddd, 13.8, 10.0, 2.2)		2.38 (brdd, 13.4, 4.6)				
7	3.93 (brd, 11.1)	71.2	4.86 (brdd, 7.9, 1.2)	119.5	2.21 m, 2.39 m	48.2	51.1
8		154.0		136.0		78.7	79.4
9	2.07 m	33.4	1.98 (dd, 15.6, 7.6)	34.3	2.11 m, 2.35 m	43.1	41.7
	2.19 m		2.09 (ddd, 15.6, 13.1, 3.1)				
10	2.07 m	24.8	1.52 (m)	34.2	5.11 s	78.3	79.0
	2.19 m		1.66 (m)				
11	5.02 (brd, 8.3)	124.4	3.90 (dd, 7.6, 5.2)	71.2	7.20 s	150.7	151.6
12		136.9		154.5		131.2	131.1
13	1.80 m	40.7	1.66 (m)	34.1	1.92 m, 2.59 m	20.8	20.1
	2.19 m		2.14 (ddd, 12.8, 12.8, 4.9)				
14	0.67 (td, 10.9, 4.0)	31.5	1.13 (dddd, 13.4, 12.8, 11.9, 3.4)	26.1	1.29 m, 1.75 m	27.6	29.2
			1.55 (dddd, 14.0, 13.4, 4.9, 2.7)				
15		20.1		20.1		145.8	145.4
16	1.00 s	15.6	1.02 s	15.5	4.67 s, 4.79 s	113.1	113.0
17	1.06 s	28.7	1.06 s	29.1	1.64 s	25.3	27.8
18	1.74 brs	10.5	1.71 (d, 1.2)	10.2	1.30 s	18.1	18.4
19	4.89 brs	109.8	1.63 (brs)	17.1		174.0	174.4
	5.04 brs						
20	1.56 brs	15.8	4.85 brs,	108.6			
			5.02 brs				

## Supplementary Materials

Supplementary materials can be accessed at: http://www.mdpi.com/1420-3049/21/3/308/s1.
