# Supplementary material for: Casbane Diterpenes from Red Sea Coral Sinularia polydactyla
Source: Molecules. 2016 Mar 3;21(3):308. doi: 10.3390/molecules21030308 (PMC6274415; doi:10.3390/molecules21030308)
Supplement: Supplementary file 1 [file molecules-21-00308-s001.pdf]

Supplementary Materials: Casbane Diterpenes from Red Sea Coral *Sinularia polydactyla*

Mohamed-Elamir F. Hegazy, Tarik A. Mohamed, Abdelsamed I. Elshamy, Montaser A. Al-Hammady, Shinji Ohta and Paul W. Paré

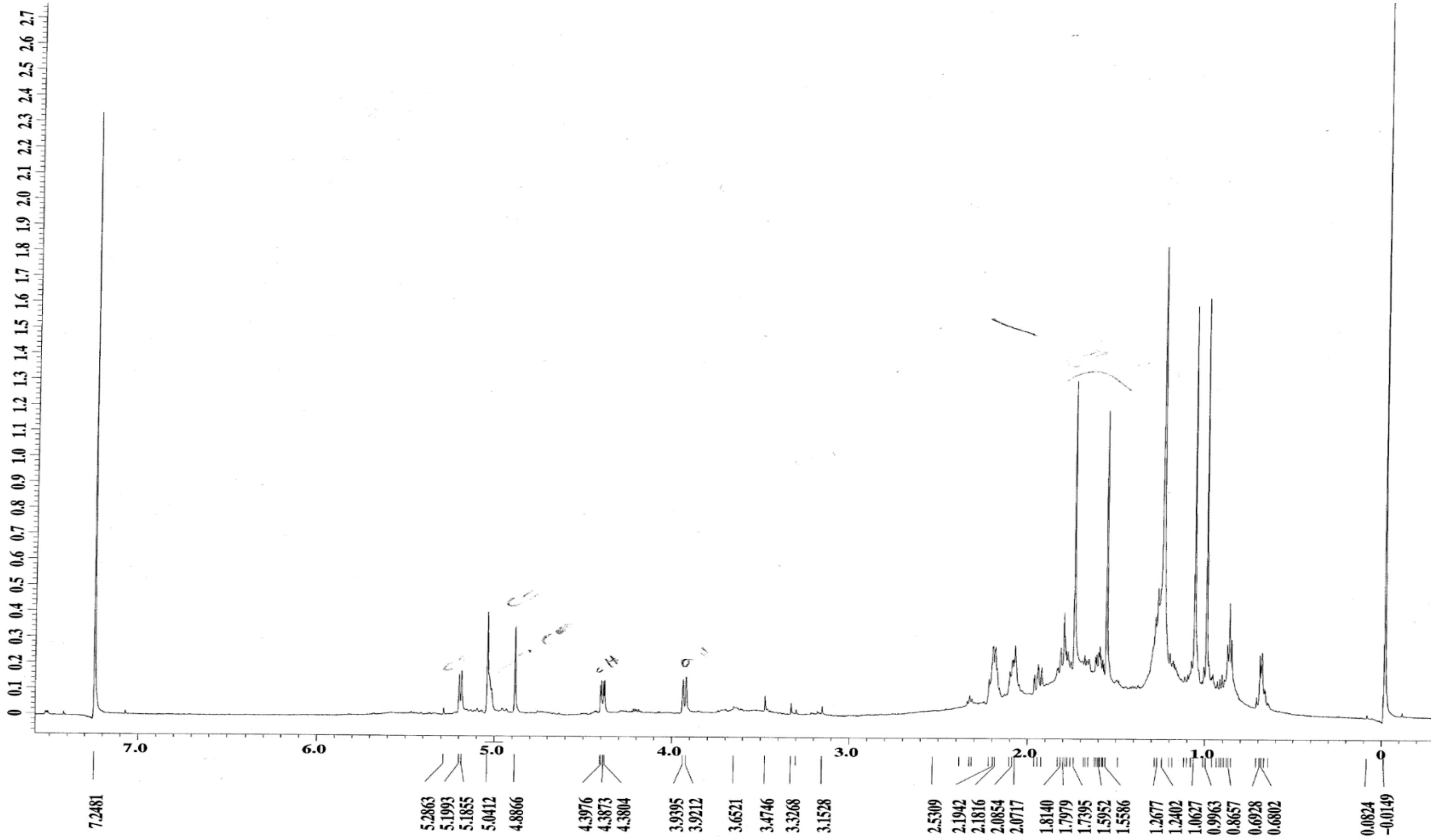

Figure S1. ^{1}H-NMR spectrum of SP-4-3-9 (Sinularcasbane G) (**1**) in $CDCl_3$.

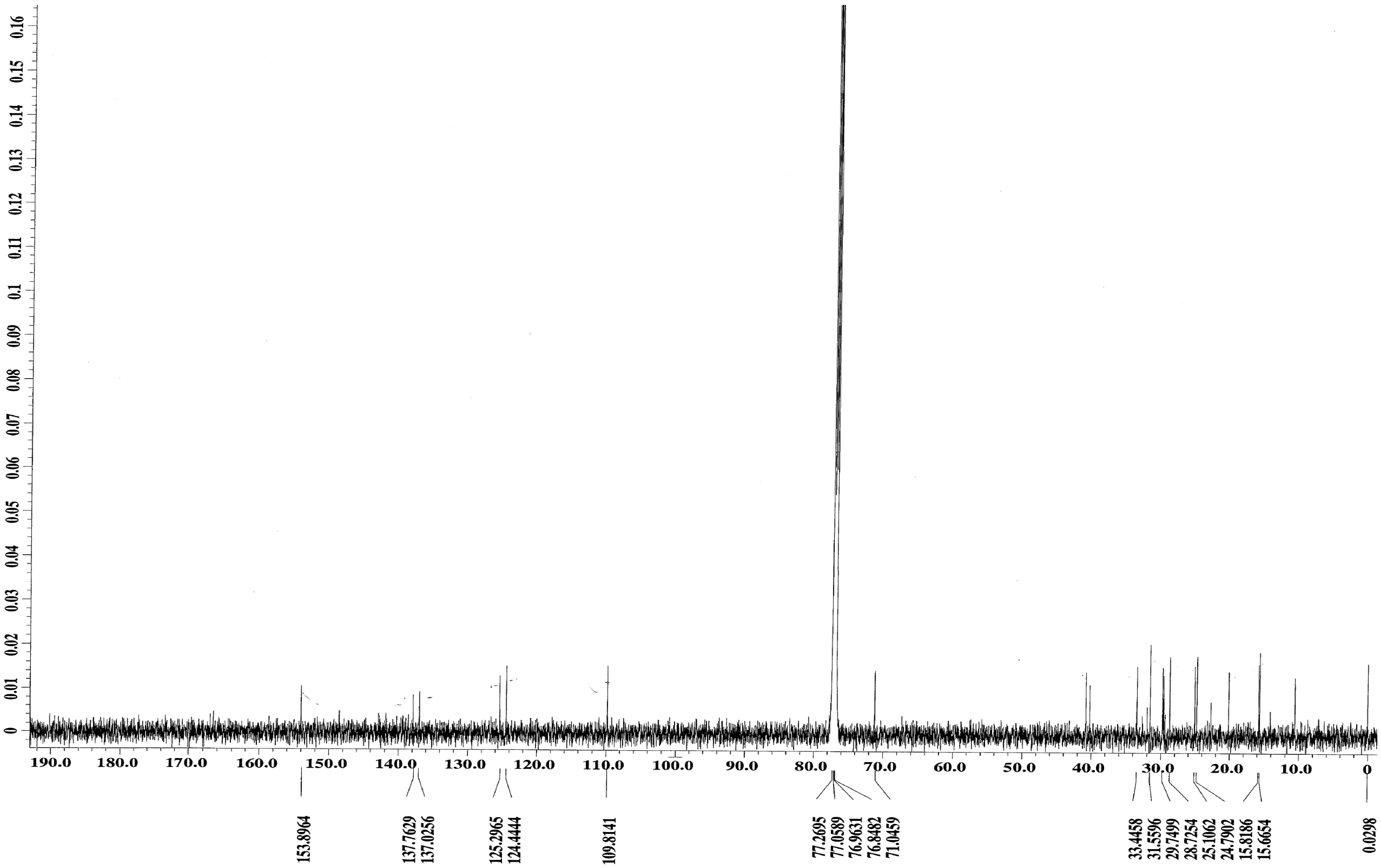

Figure S2. ^{13}C-NMR spectrum of SP-4-3-9 (Sinularcasbane G) (**1**) in $CDCl_3$.

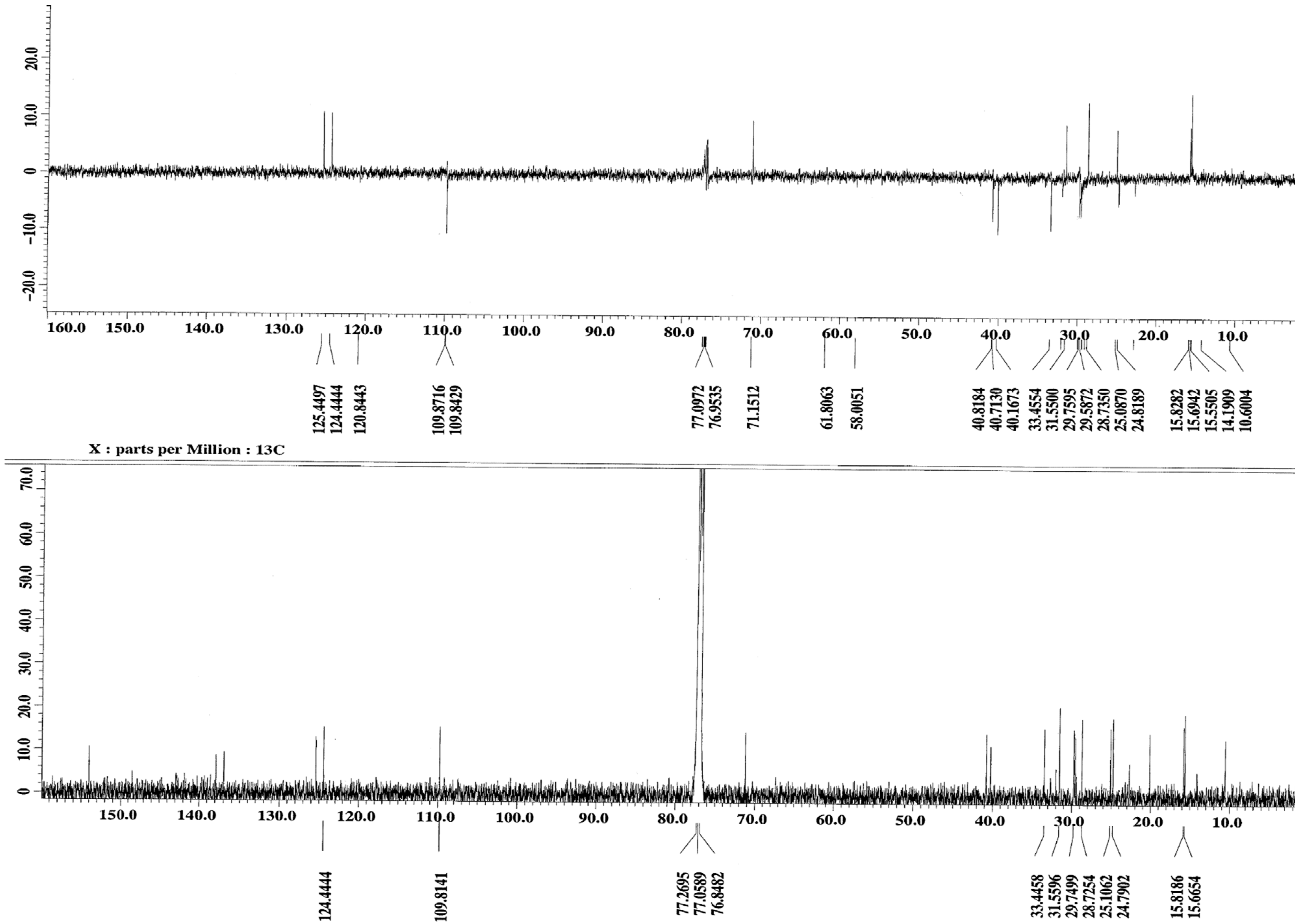

Figure S3. DEPT spectrum of SP-4-3-9 (Sinularcasbane G) (**1**) in $CDCl_3$.

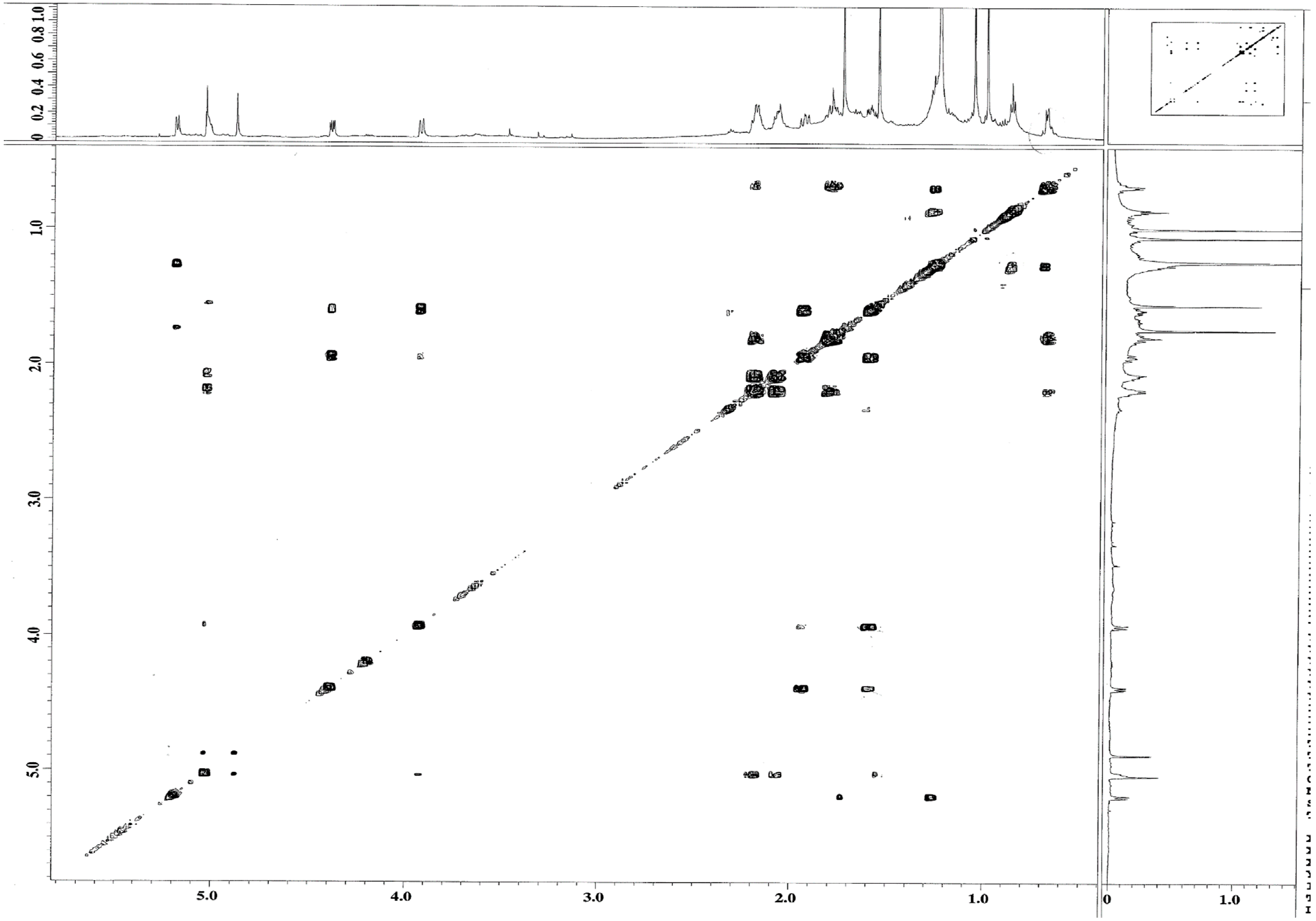

Figure S4. ^{1}H-^{1}H COSY spectrum of SP-4-3-9 (Sinularcasbane G) (**1**) in $CDCl_3$.

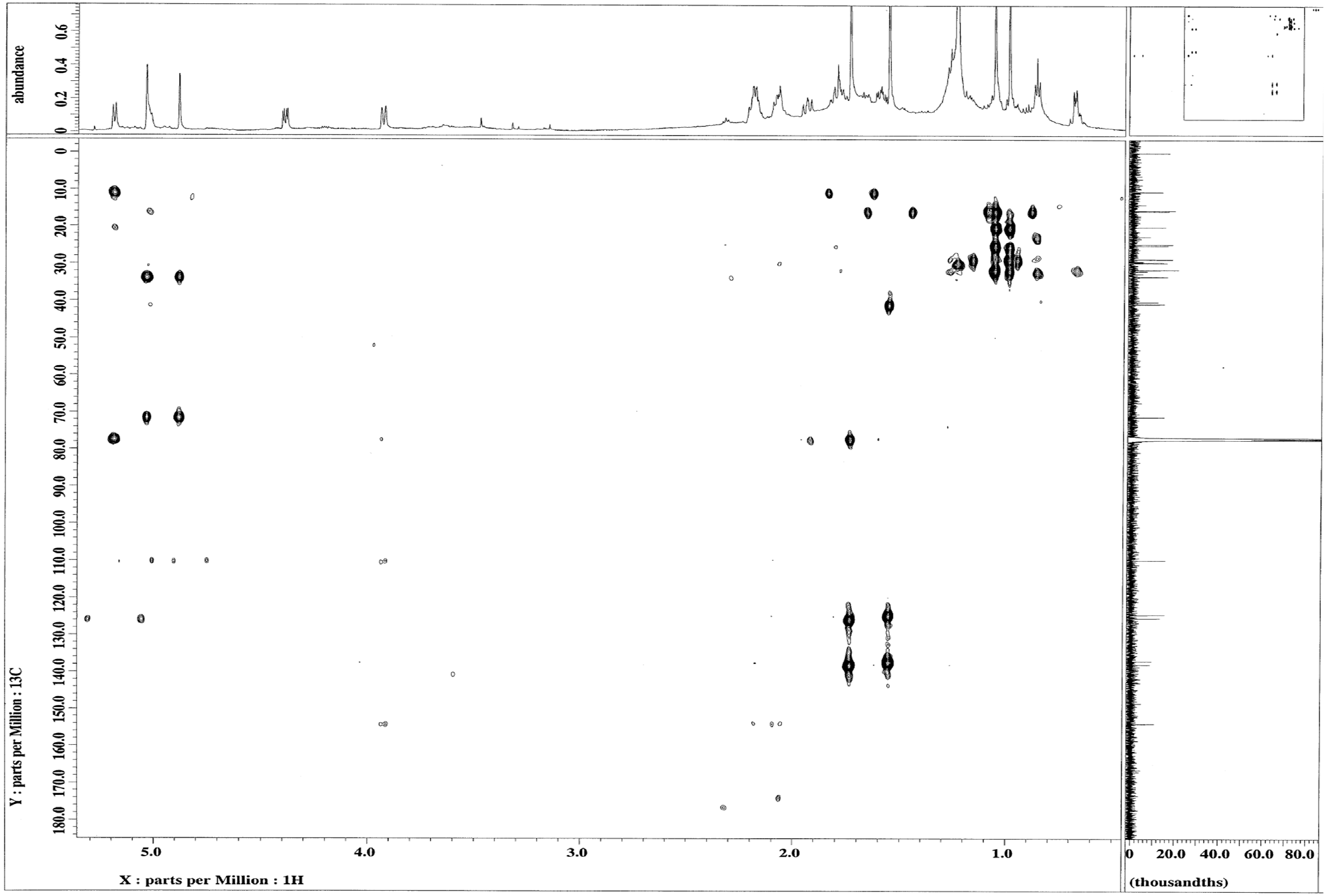

Figure S5. HMQC spectrum of SP-4-3-9 (Sinularcasbane G) (**1**) in $CDCl_3$.

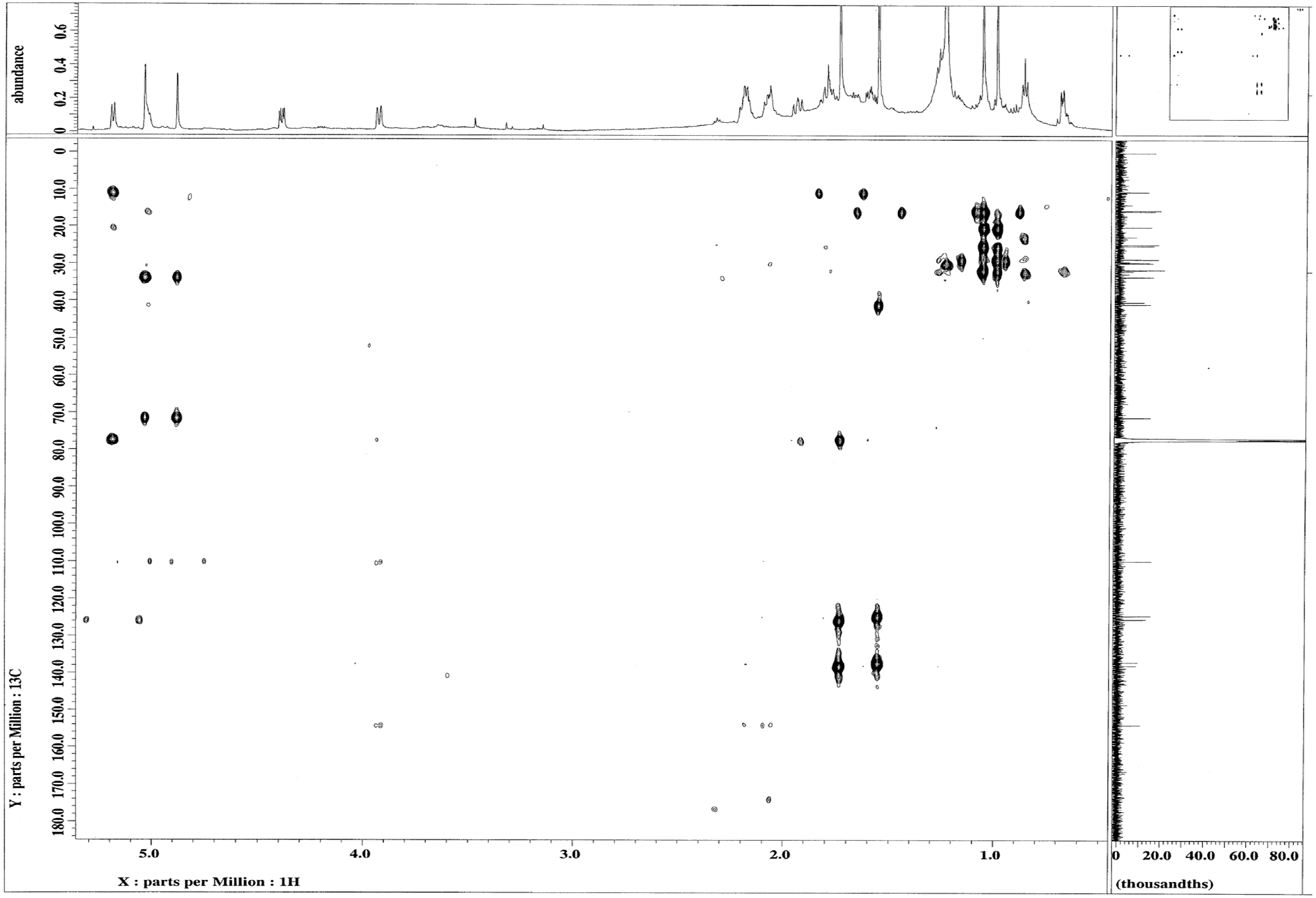

Figure S6. HMBC spectrum of SP-4-3-9 (Sinularcasbane G) (**1**) in $CDCl_3$.

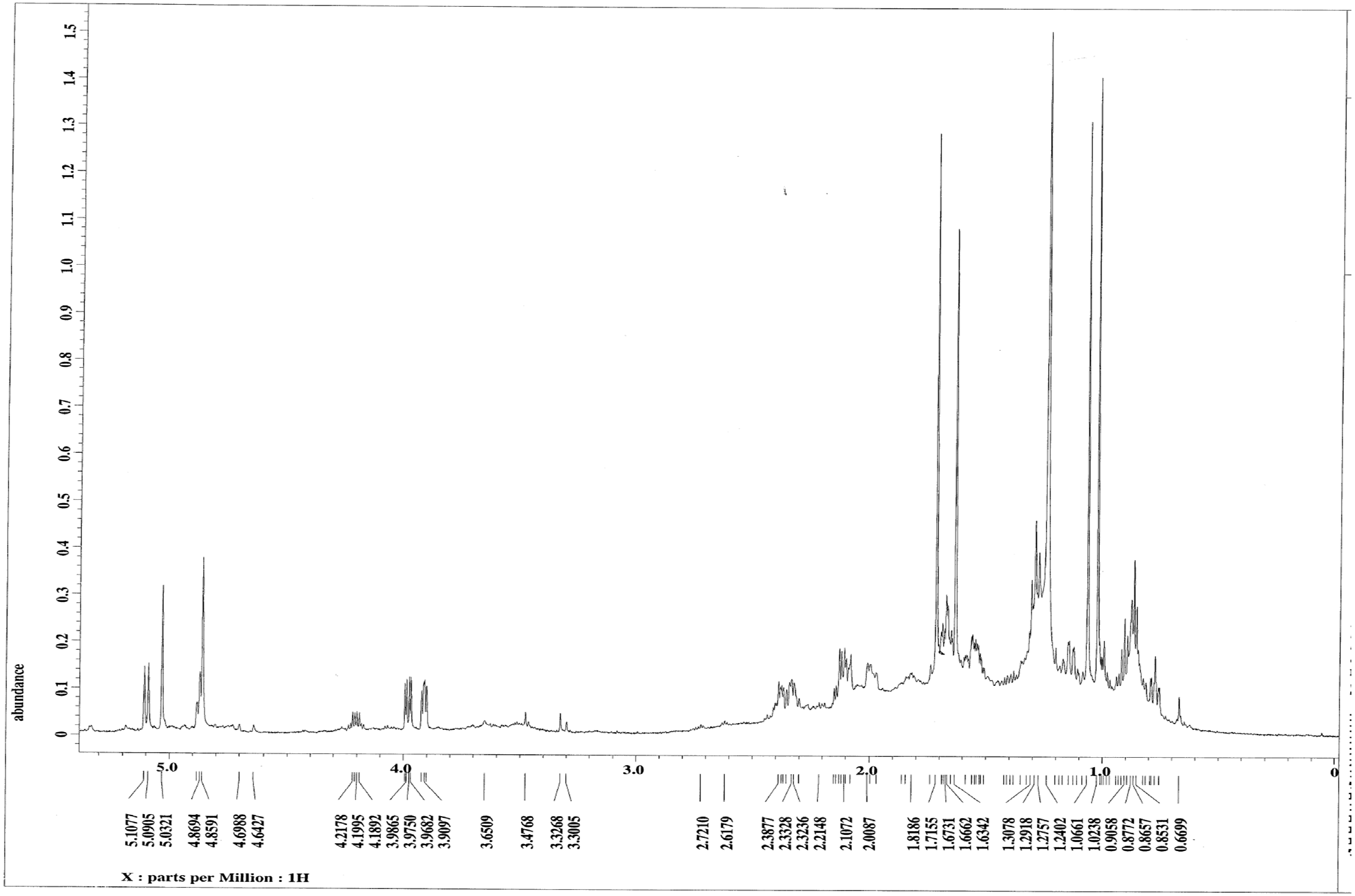

Figure S7. ^{1}H-NMR spectrum of SP-4-3-7 (Sinularcasbane H) (**2**) in $CDCl_3$.

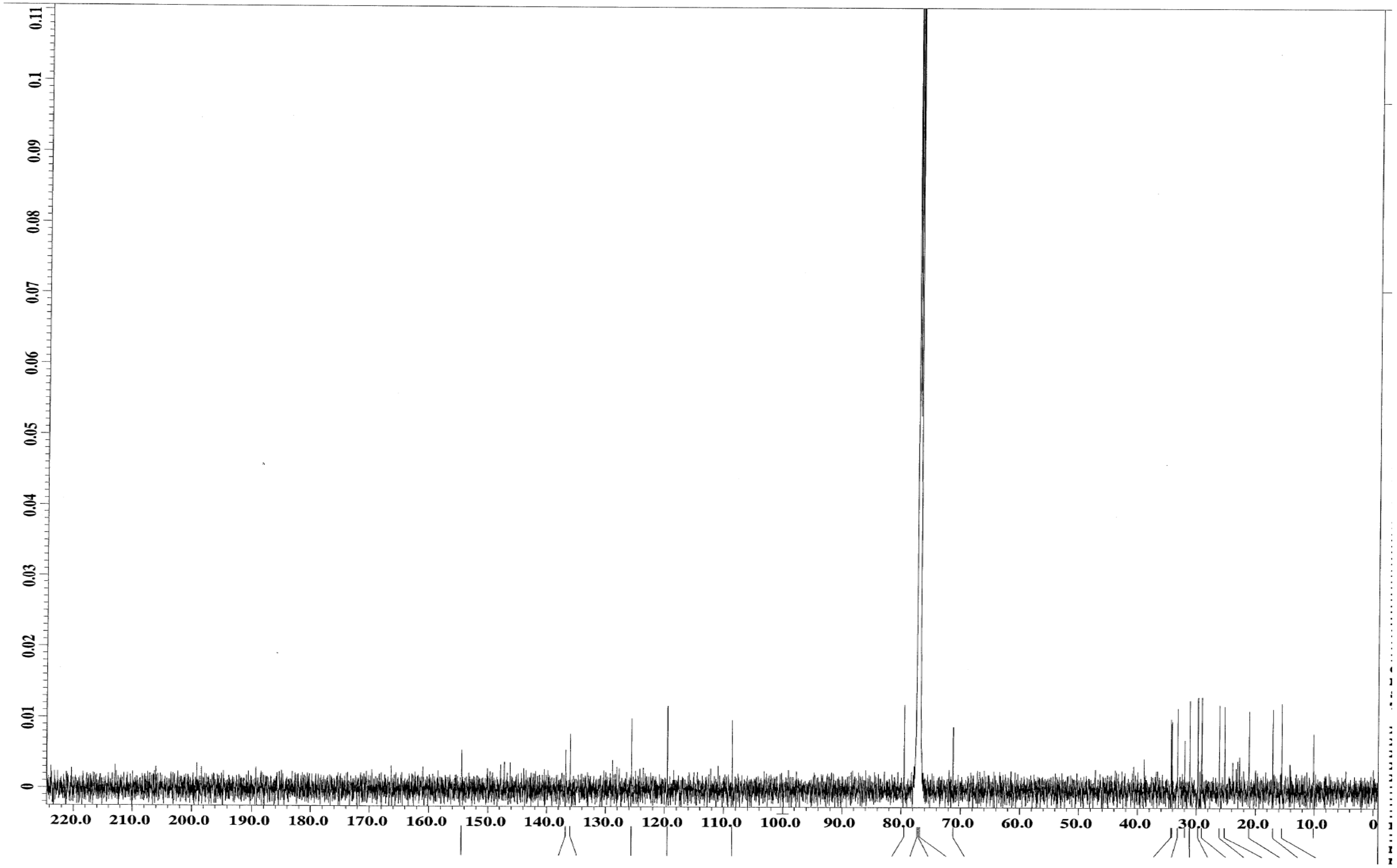

Figure S8. ^{13}C-NMR spectrum of SP-4-3-7 (Sinularcasbane H) (**2**) in $CDCl_3$.

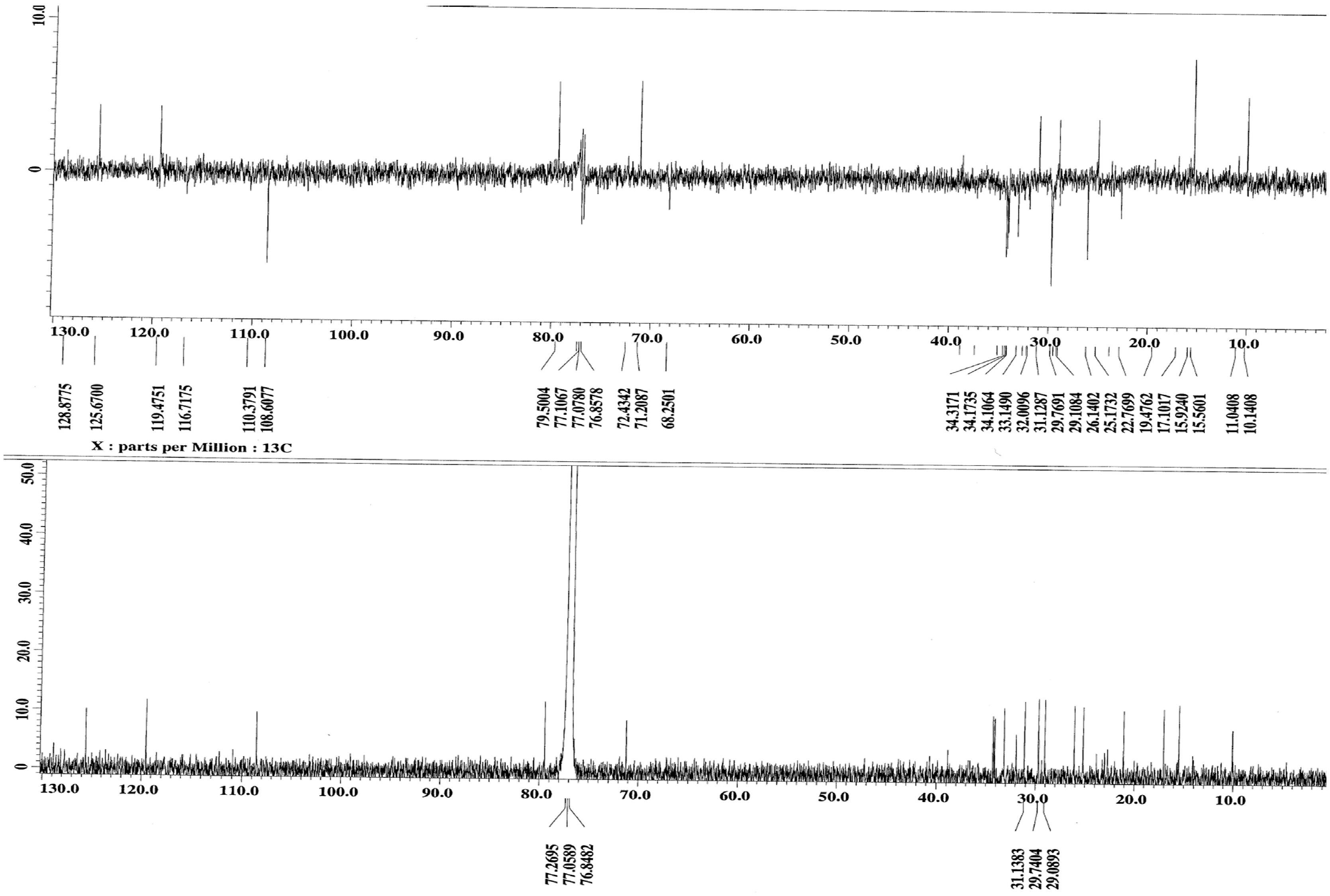

Figure S9. DEPT spectrum of SP-4-3-7 (Sinularcasbane H) (**2**) in $CDCl_3$.

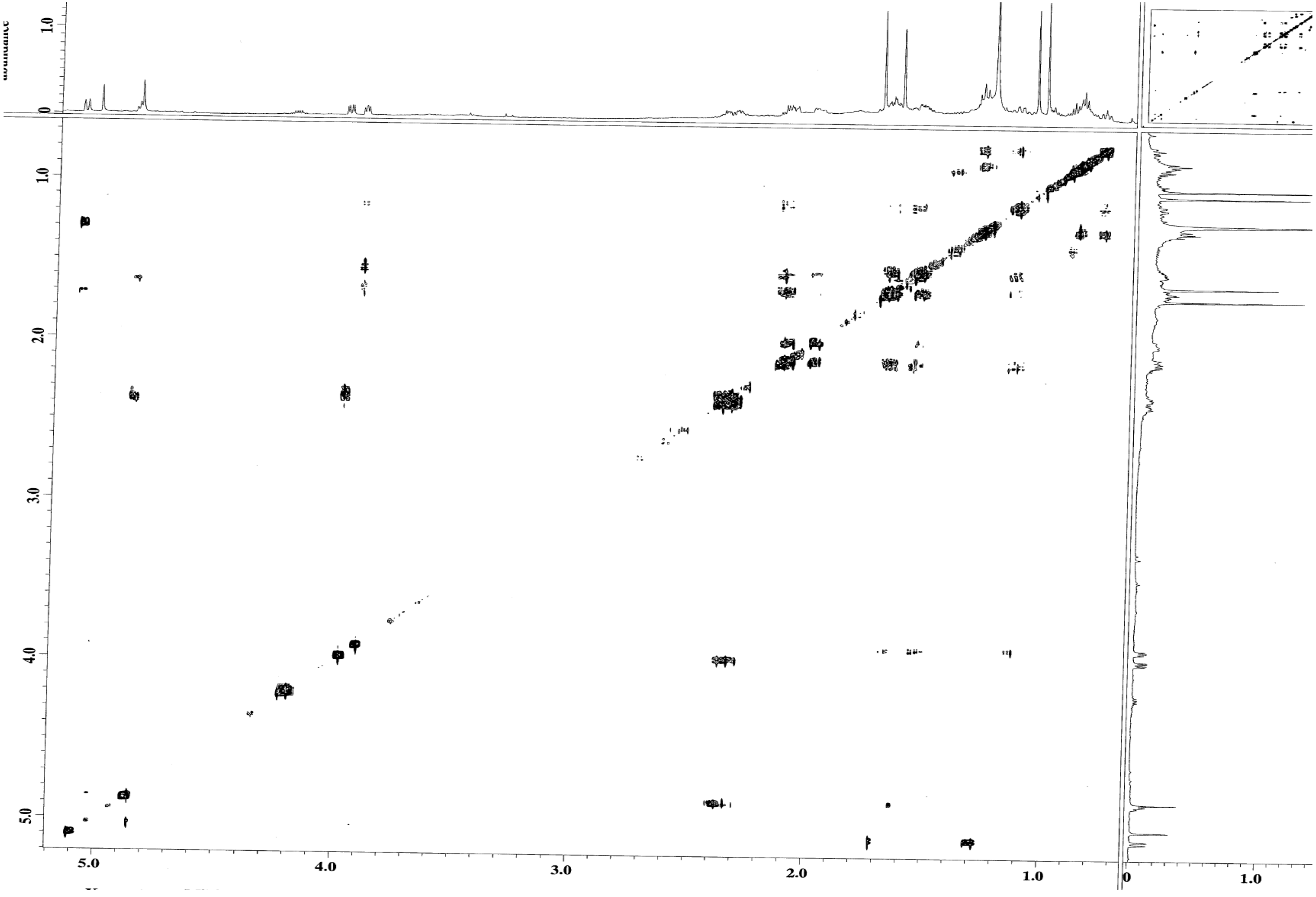

Figure S10. ^{1}H-^{1}H COSY spectrum of SP-4-3-7 (Sinularcasbane H) (**2**) in $CDCl_3$.

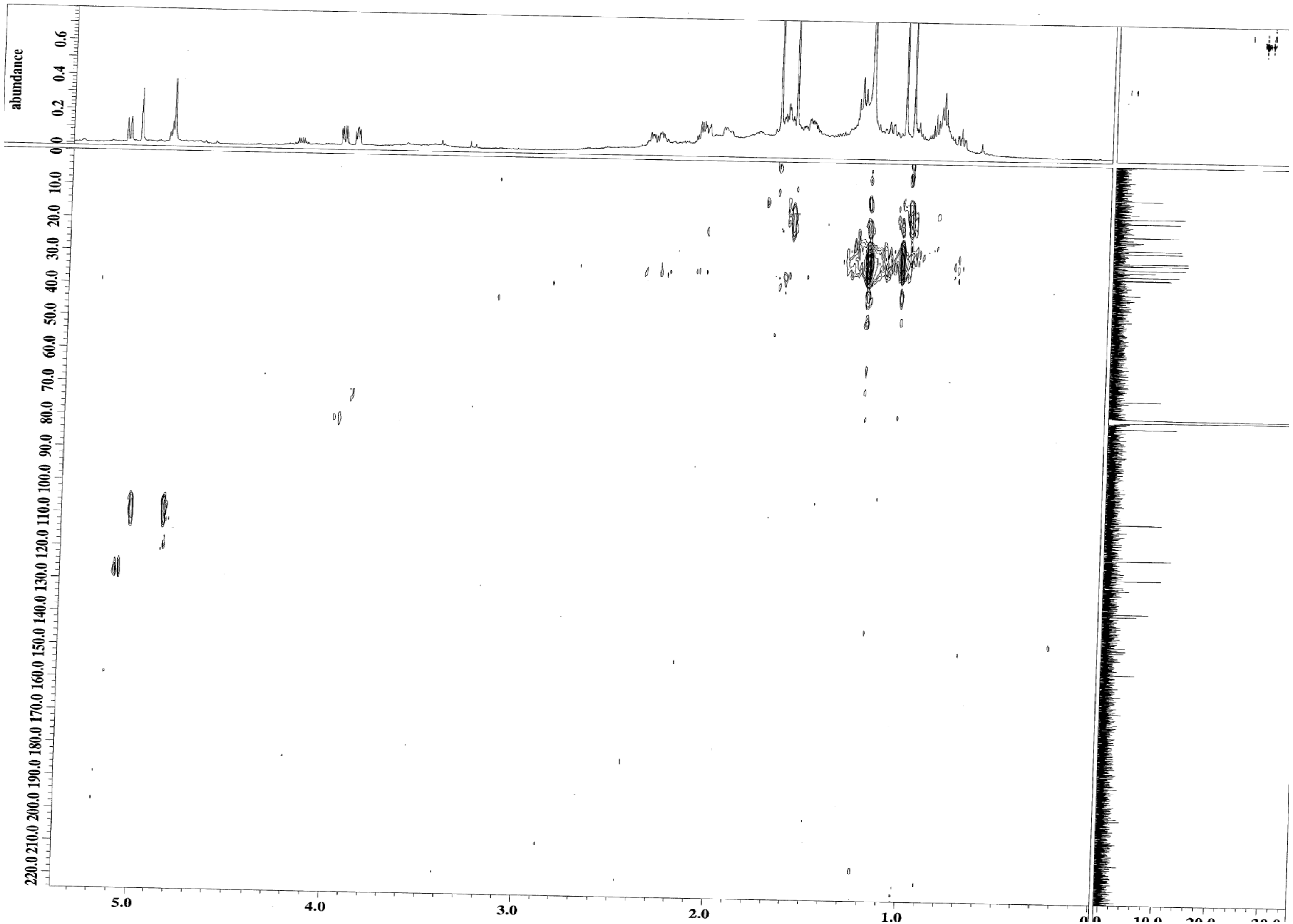

Figure S11. HMQC spectrum of SP-4-3-7 (Sinularcasbane H) (**2**) in $CDCl_3$.

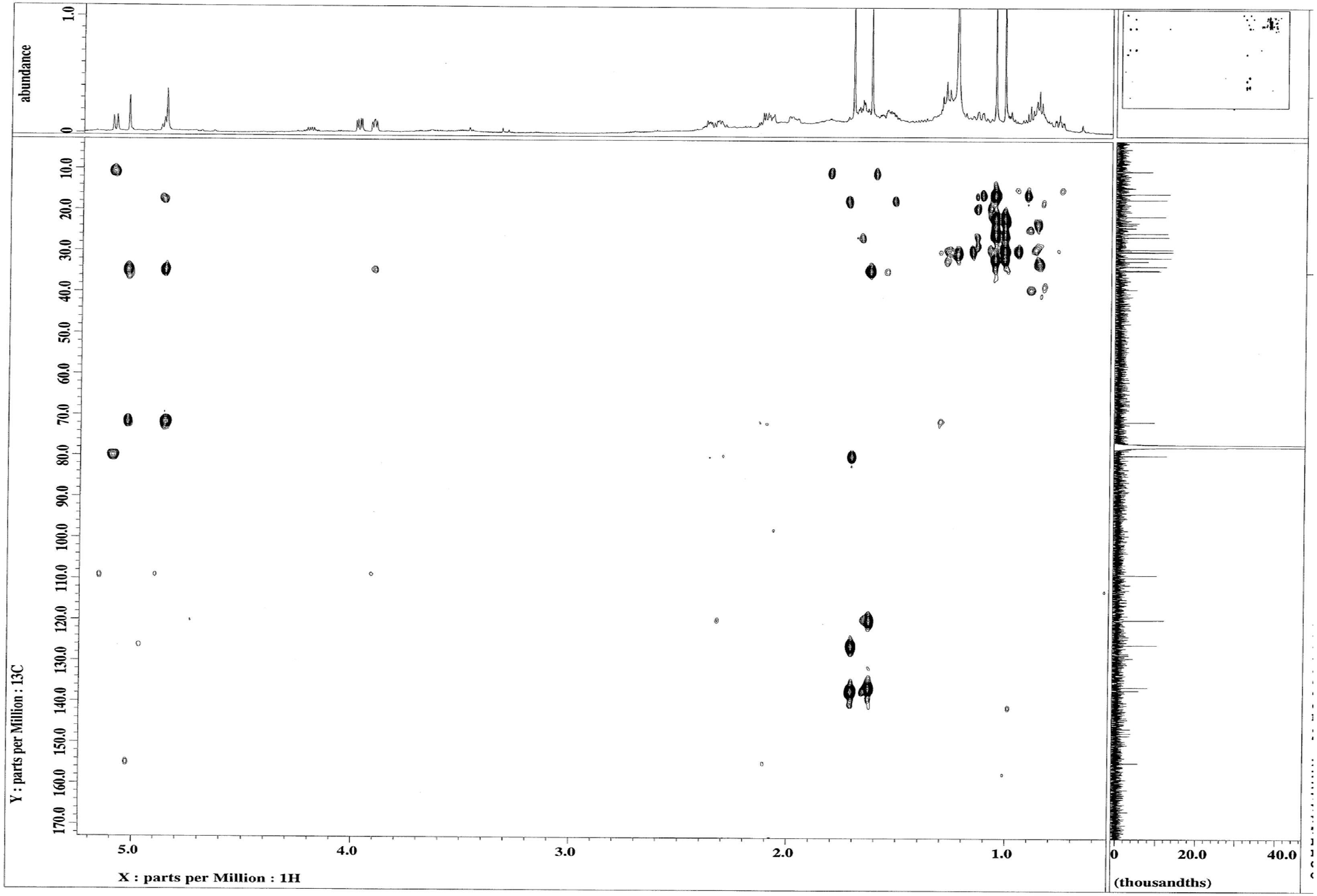

Figure S12. HMBC spectrum of SP-4-3-7 (Sinularcasbane H) (**2**) in $CDCl_3$.

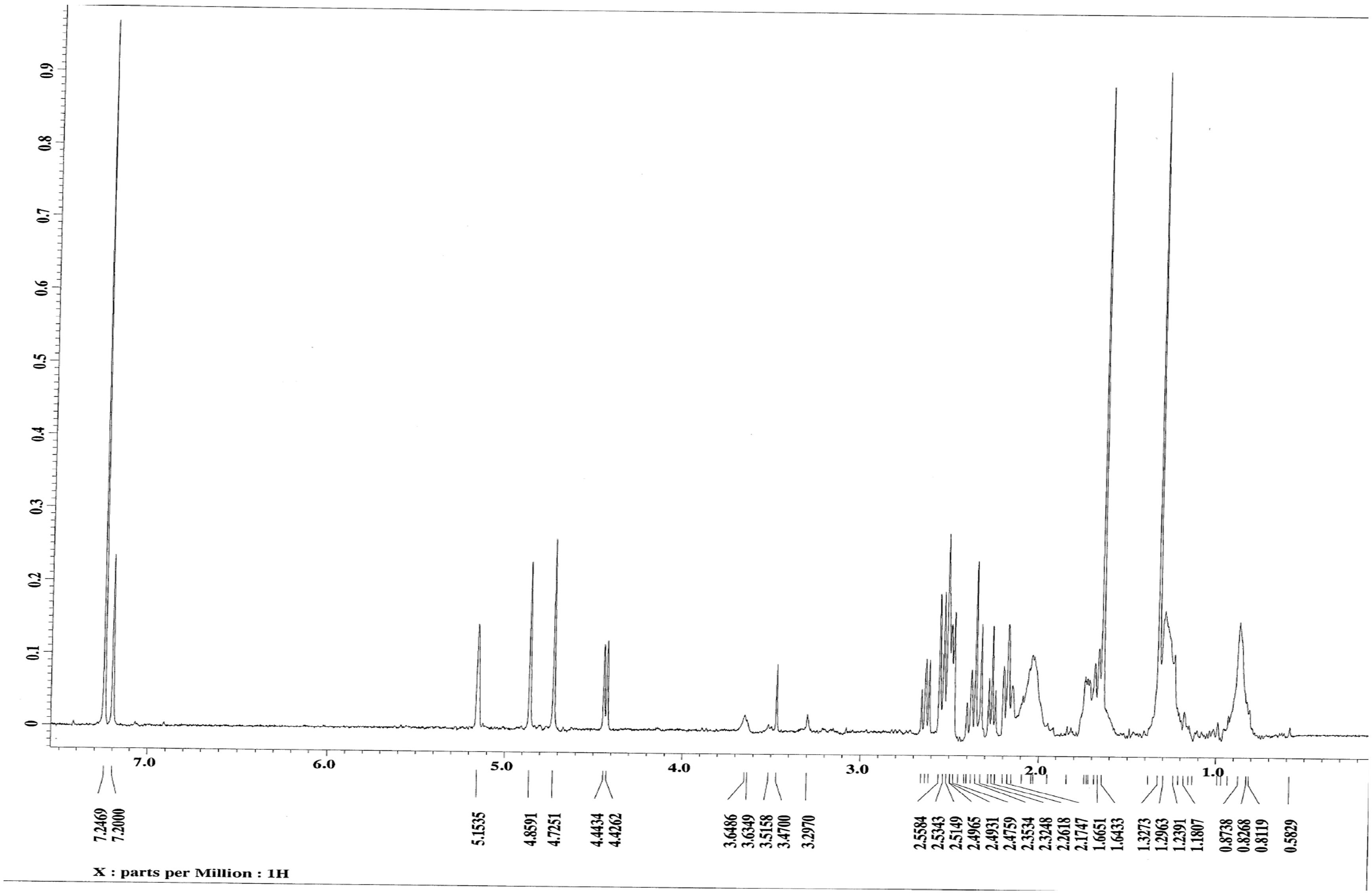

Figure S13. ^{1}H-NMR spectrum of SP-3-13-4 (5-epi-norcembrenolide) (**3**) in $CDCl_3$.

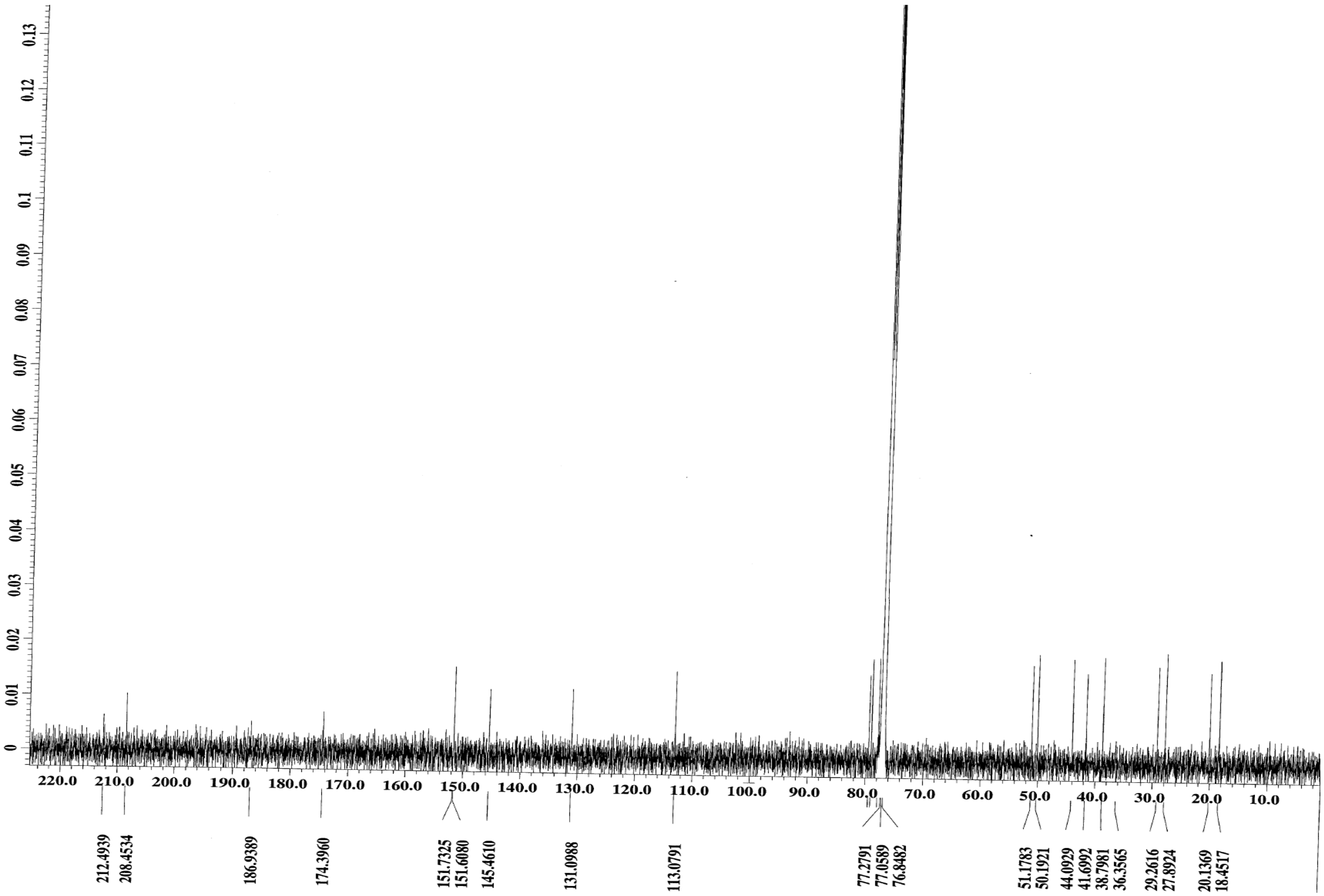

Figure S14. ^{13}C-NMR spectrum of SP-3-13-4 (5-epi-norcembrenolide) (**3**) in $CDCl_3$.

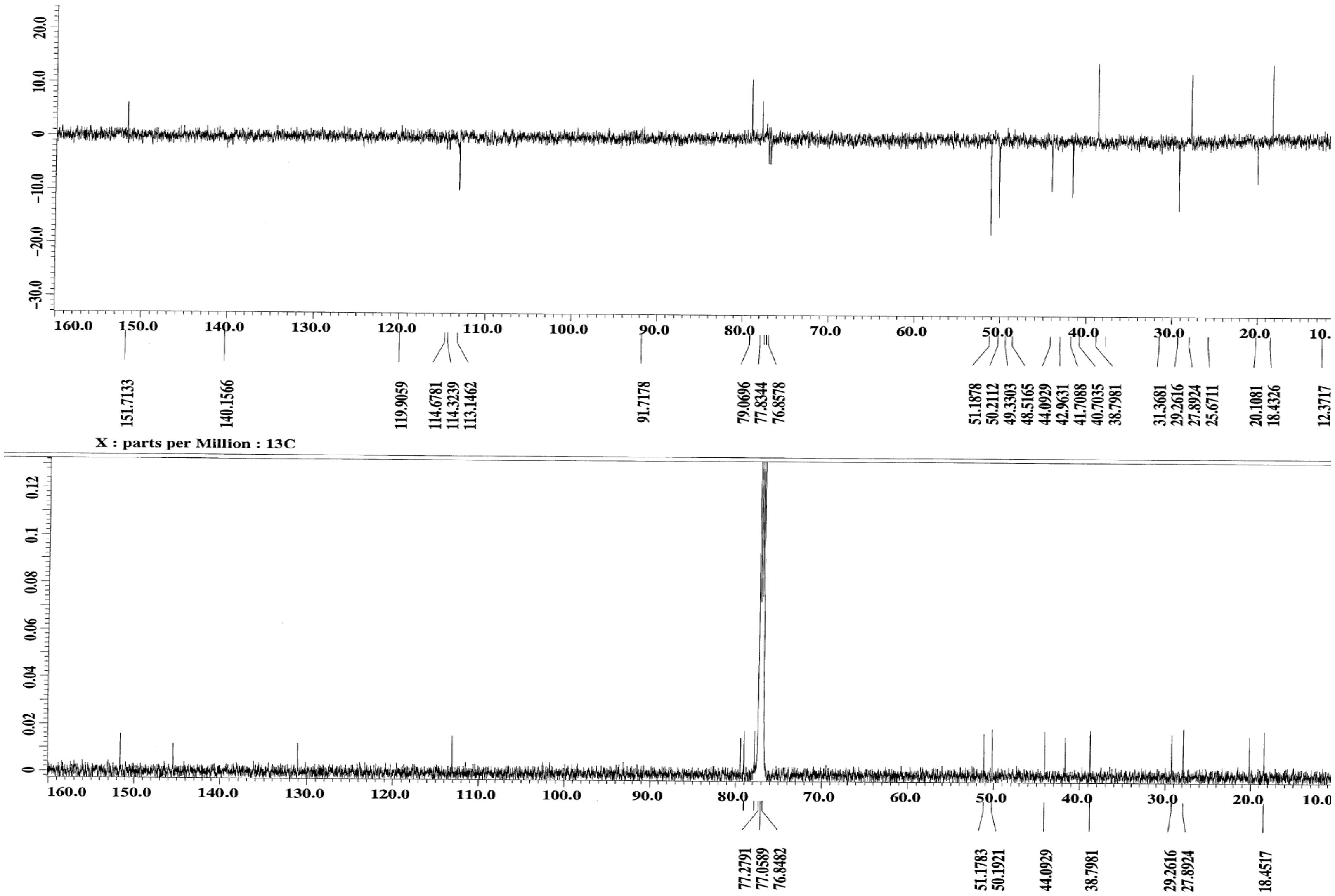

Figure S15. DEPT spectrum of SP-3-13-4 (5-epi-norcembrenolide) (**3**) in $CDCl_3$.

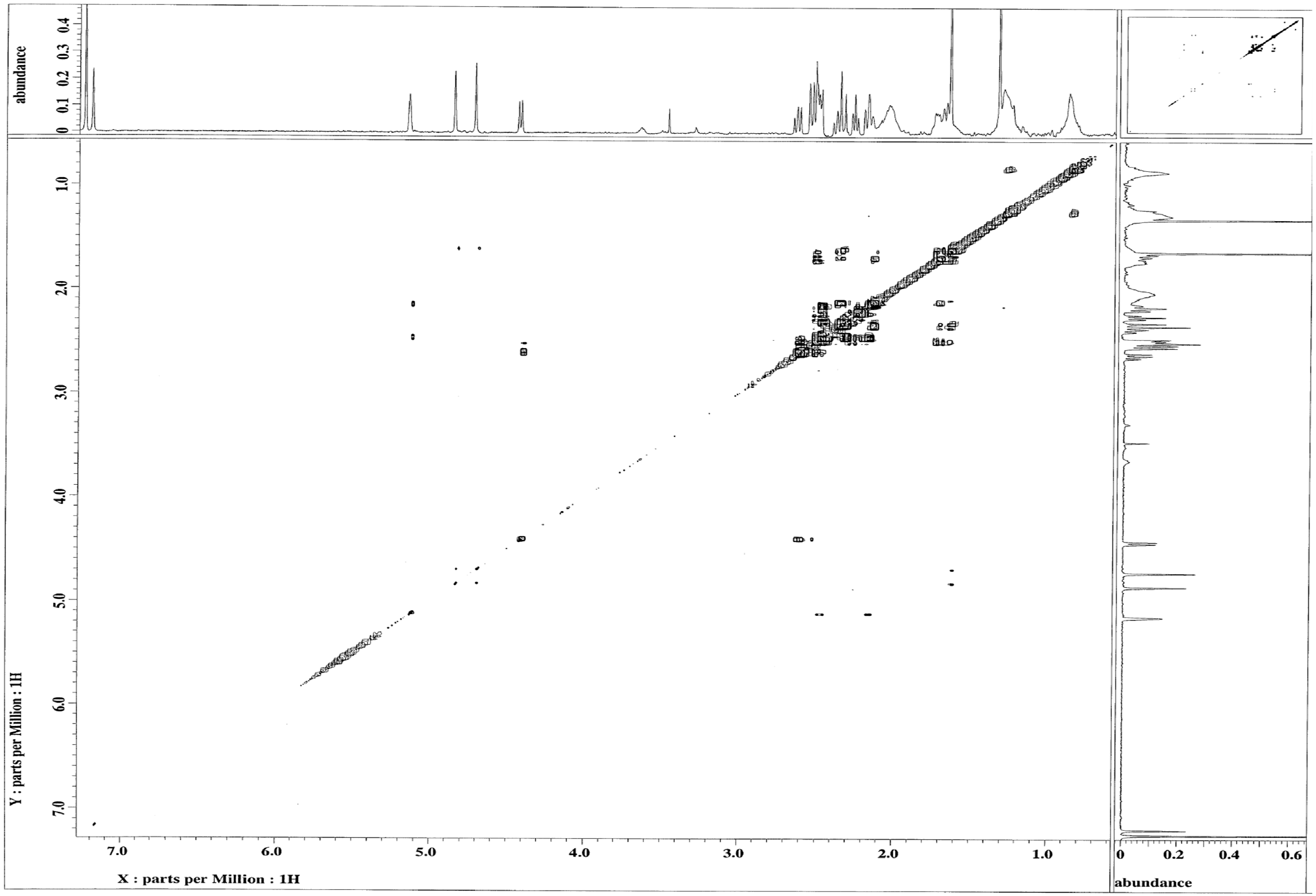

Figure S16. ^1H-^1H COSY spectrum of SP-3-13-4 (5-epi-norcembrenolide) (**3**) in $CDCl_3$.

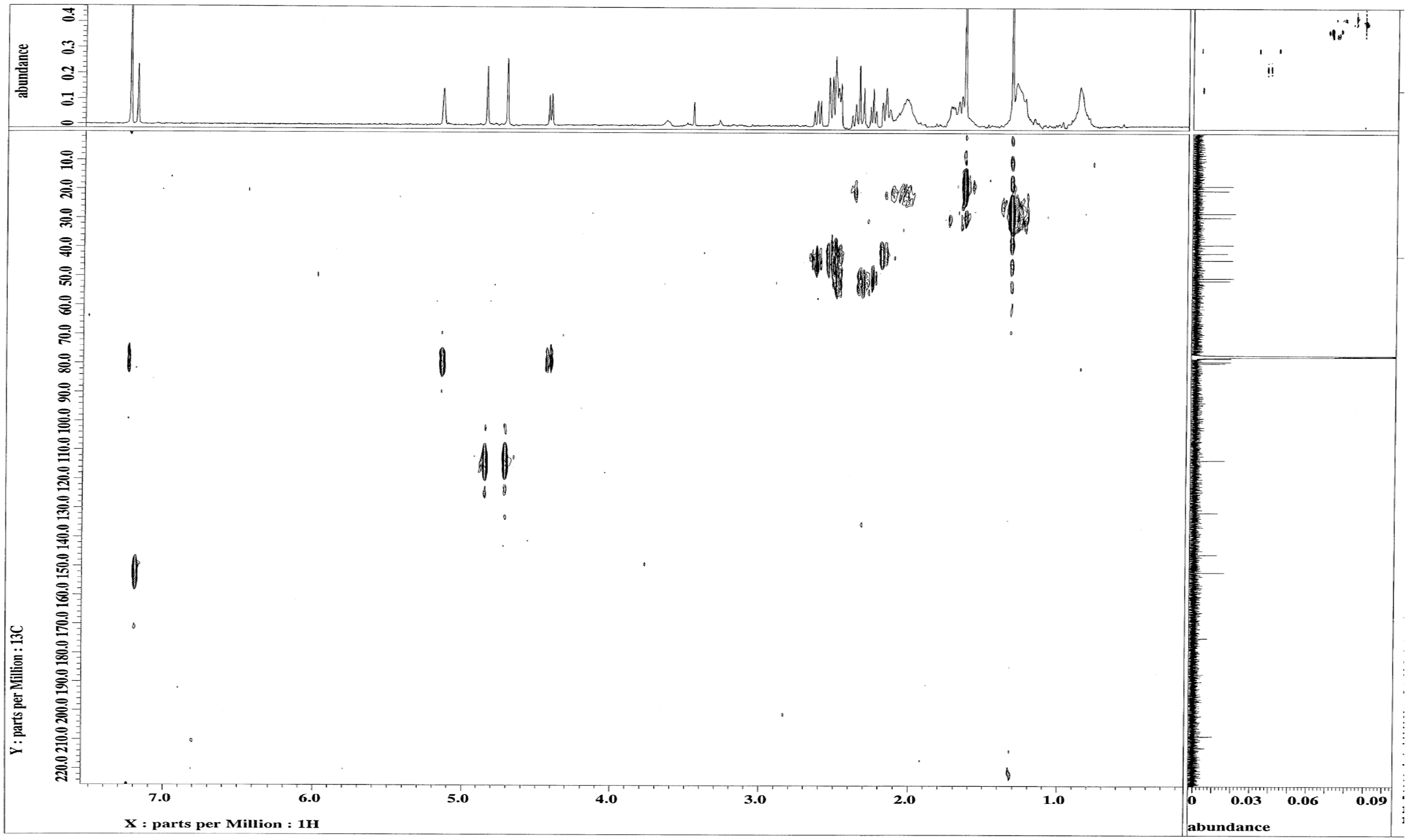

Figure S17. HMQC spectrum of SP-3-13-4 (5-epi-norcembrenolide) (**3**) in $CDCl_3$.

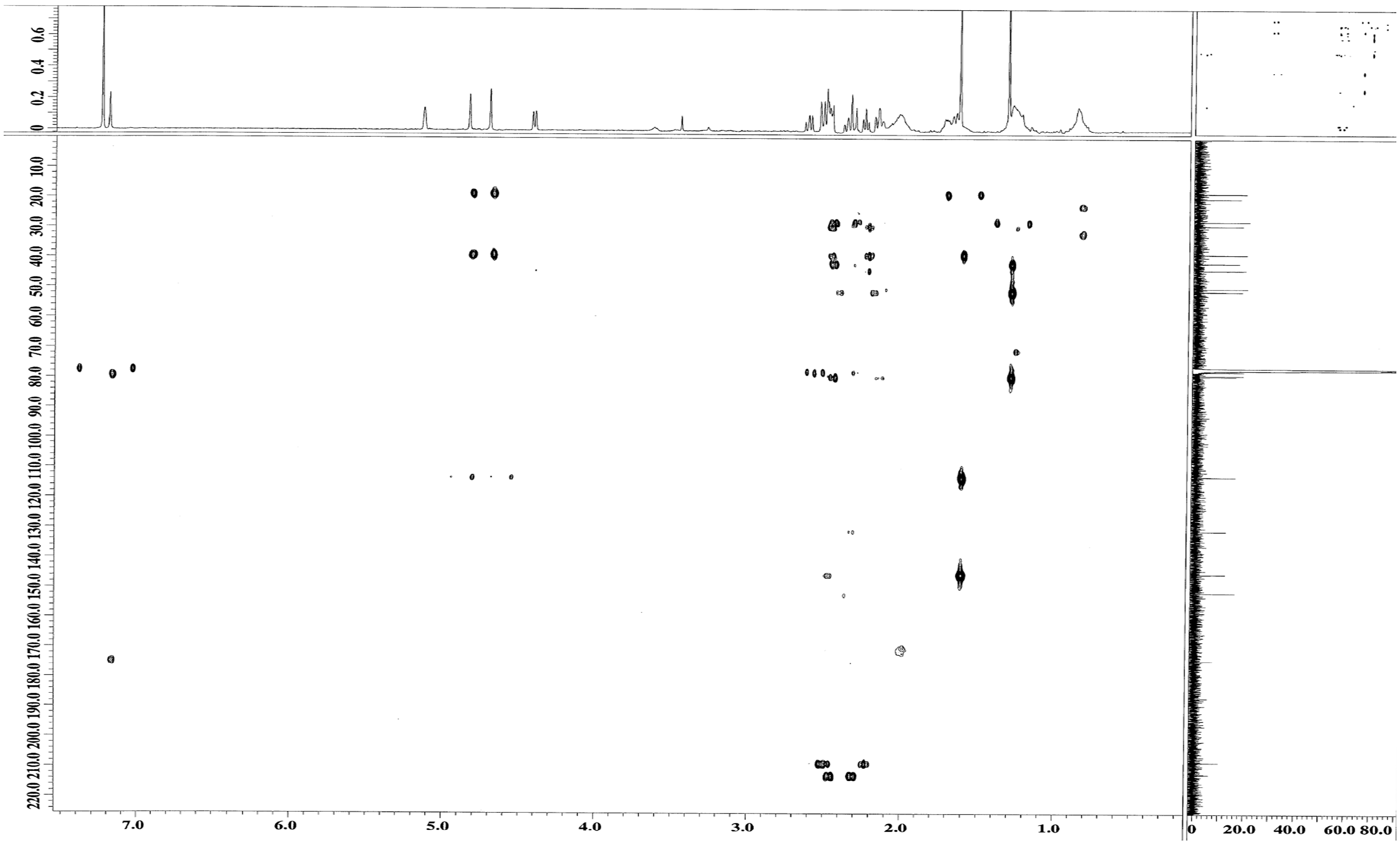

Figure S18. HMBC spectrum of SP-3-13-4 (5-epi-norcembrenolide) (**3**) in $CDCl_3$.

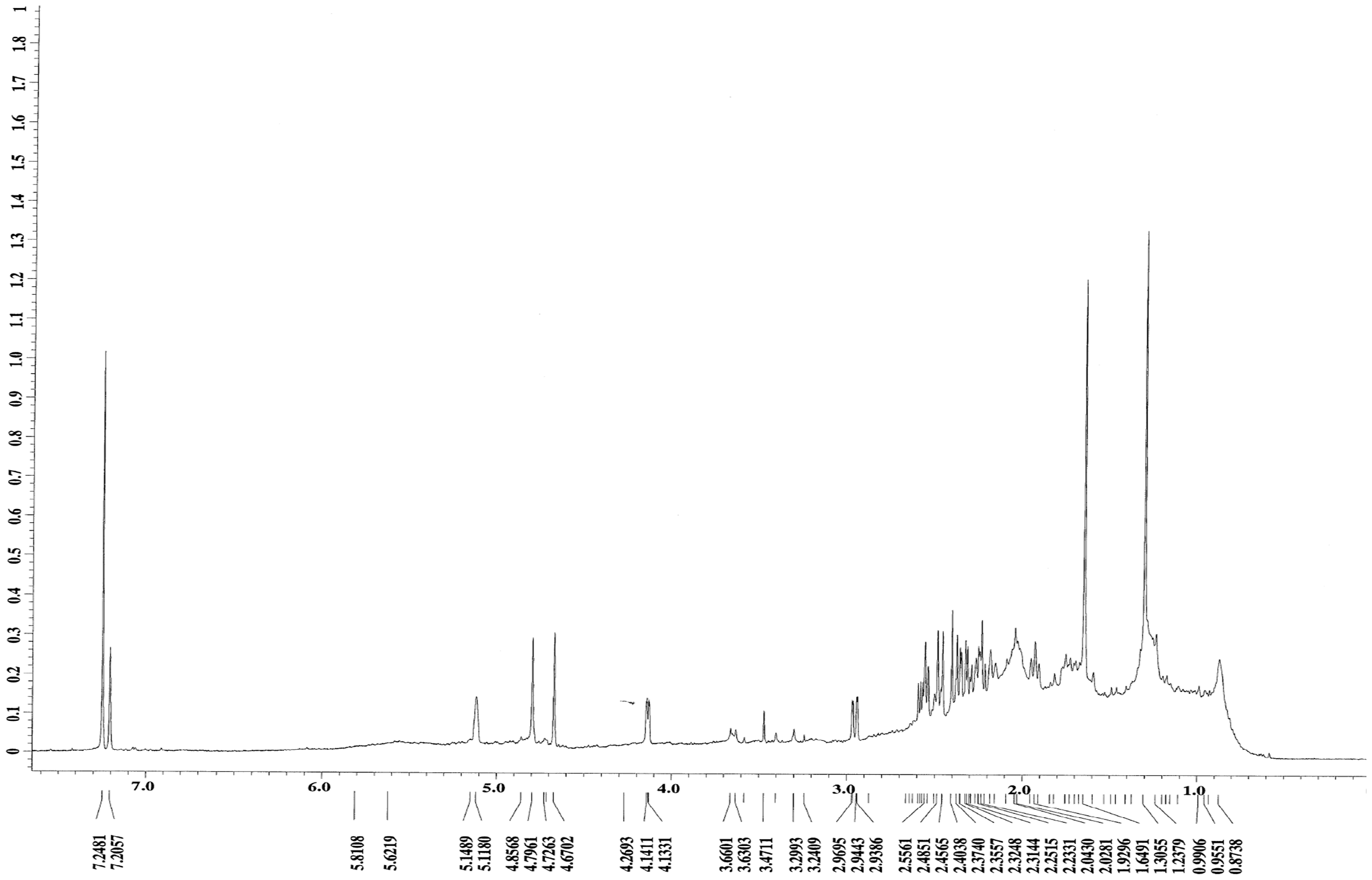

Figure S19. ^{1}H-NMR spectrum of SP-3-13-3 (norcembrenolide B) (**4**) in $CDCl_3$.

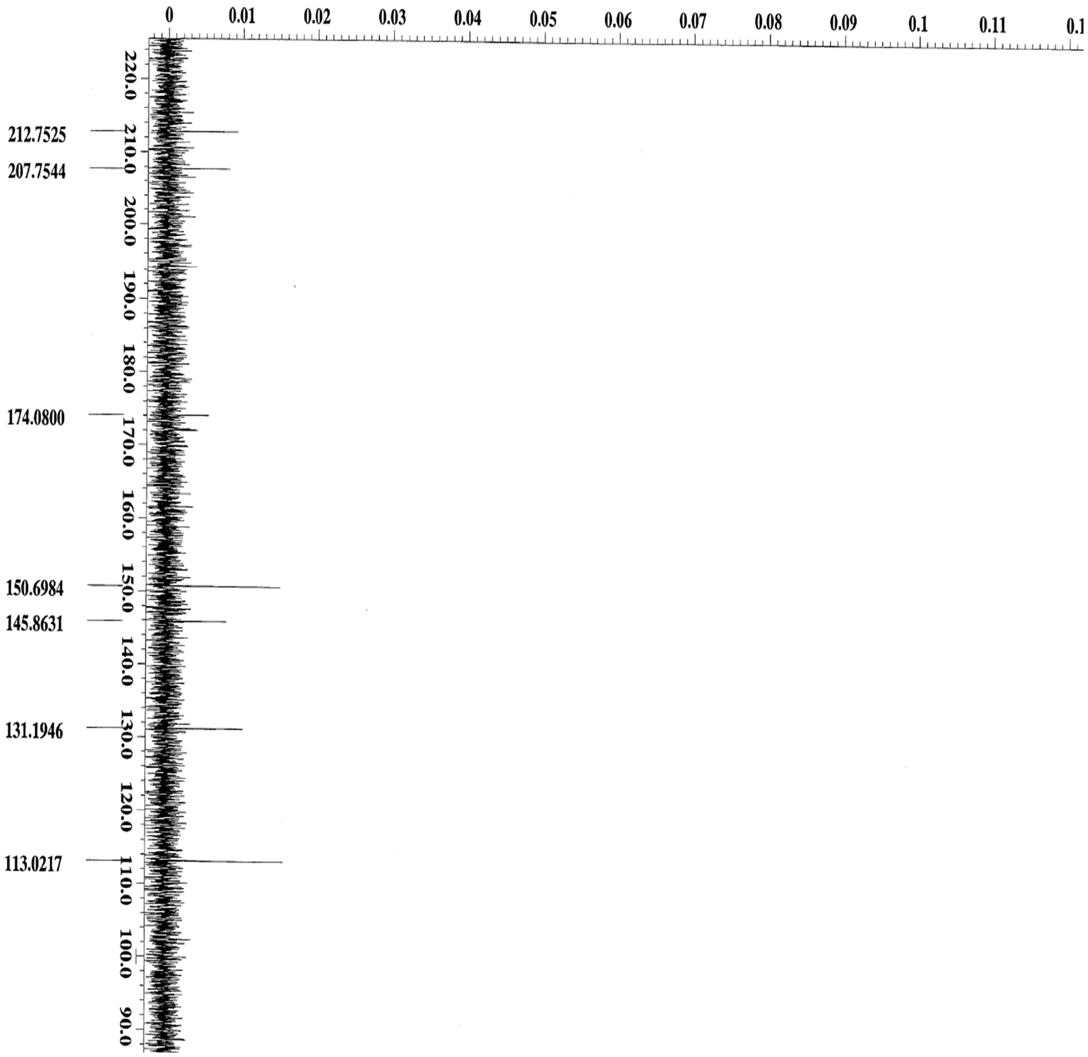

Figure S20. ^{13}C-NMR spectrum of SP-3-13-3 (norcembrenolide B) (**4**) in $CDCl_3$.

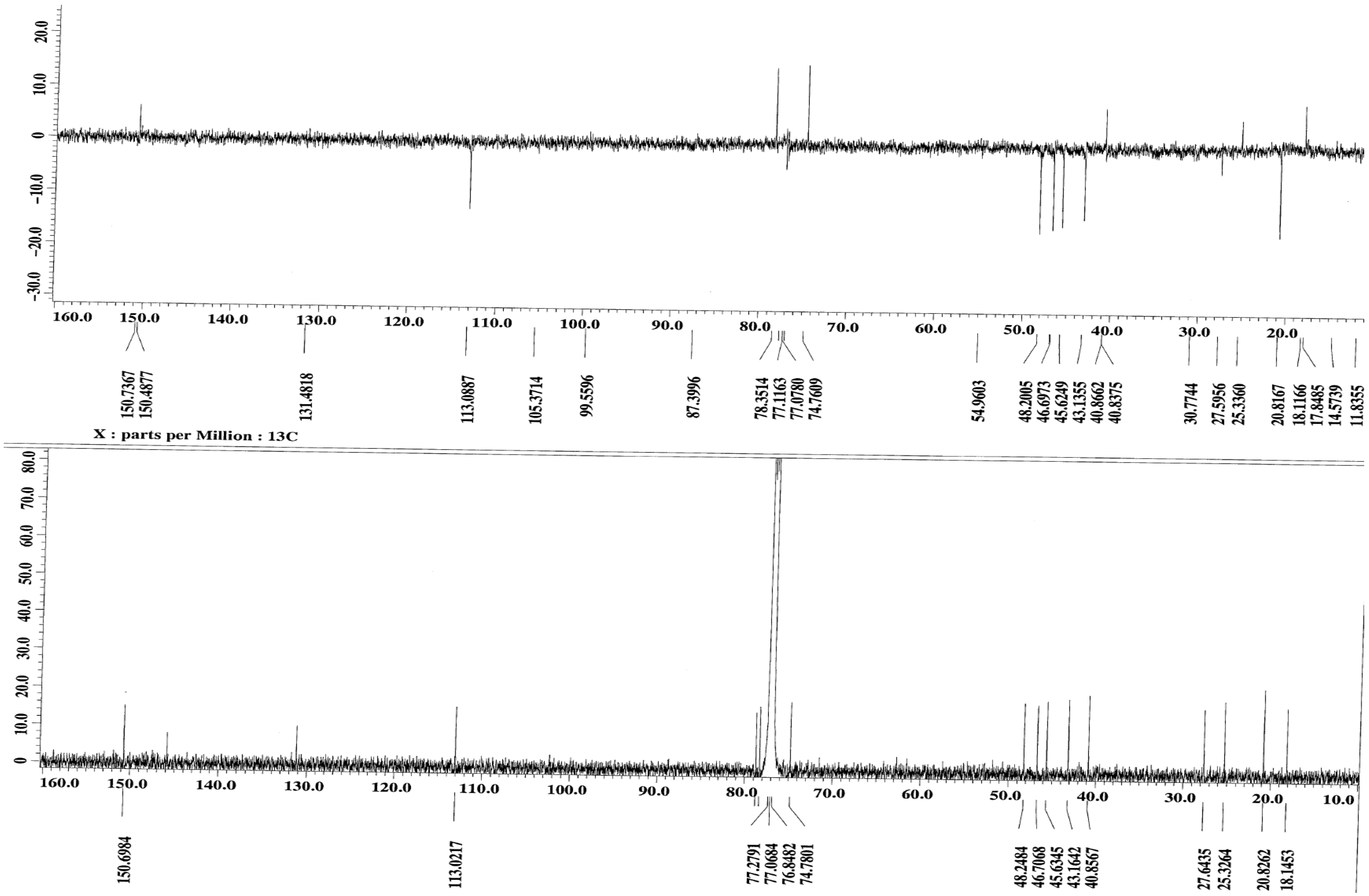

Figure S21. DEPT spectrum of SP-3-13-3 (norcembrenolide B) (**4**) in $CDCl_3$.

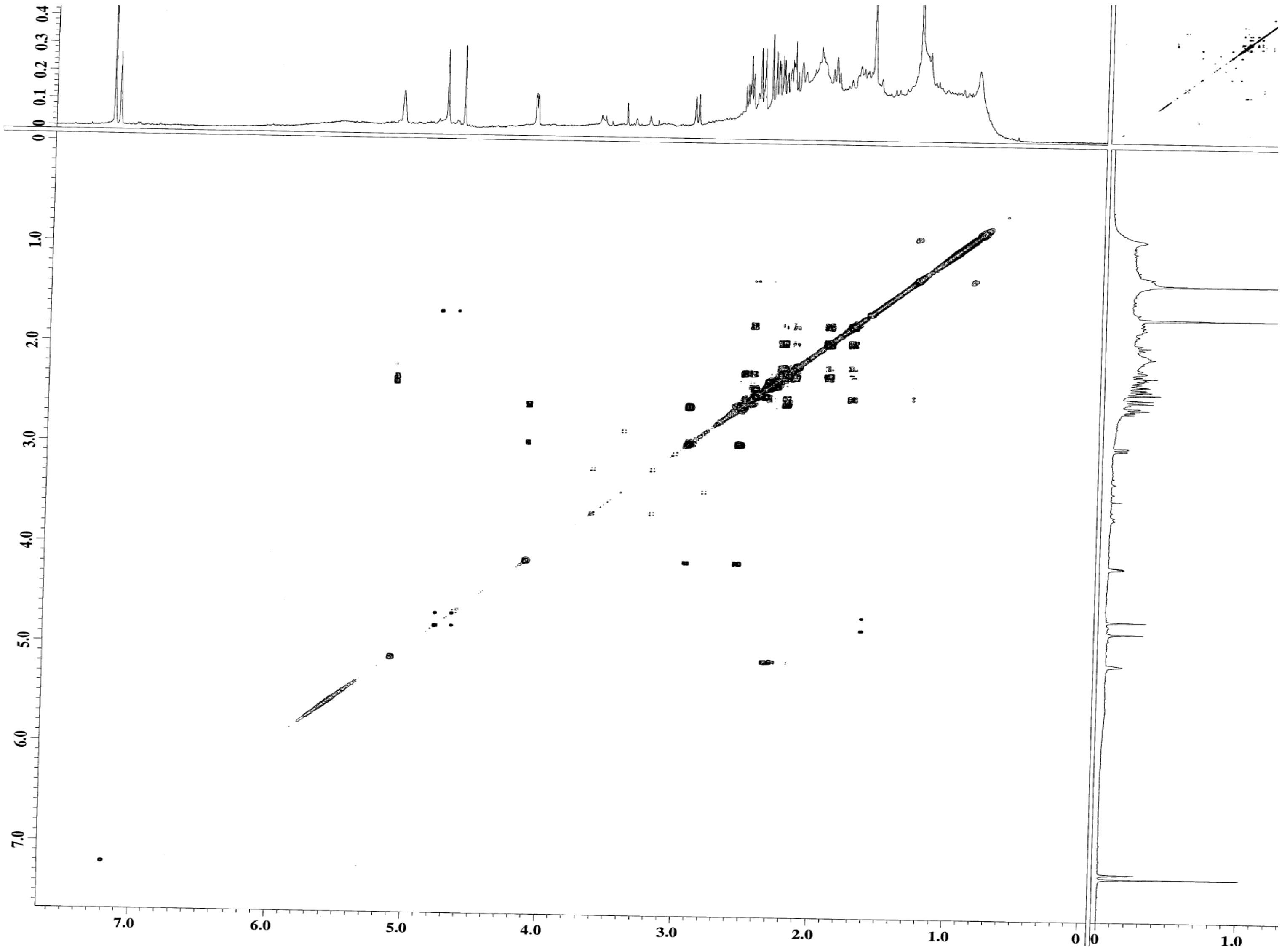

Figure S22. ^1H-^1H COSY spectrum of SP-3-13-3 (norcembrenolide B) (**4**) in $CDCl_3$.

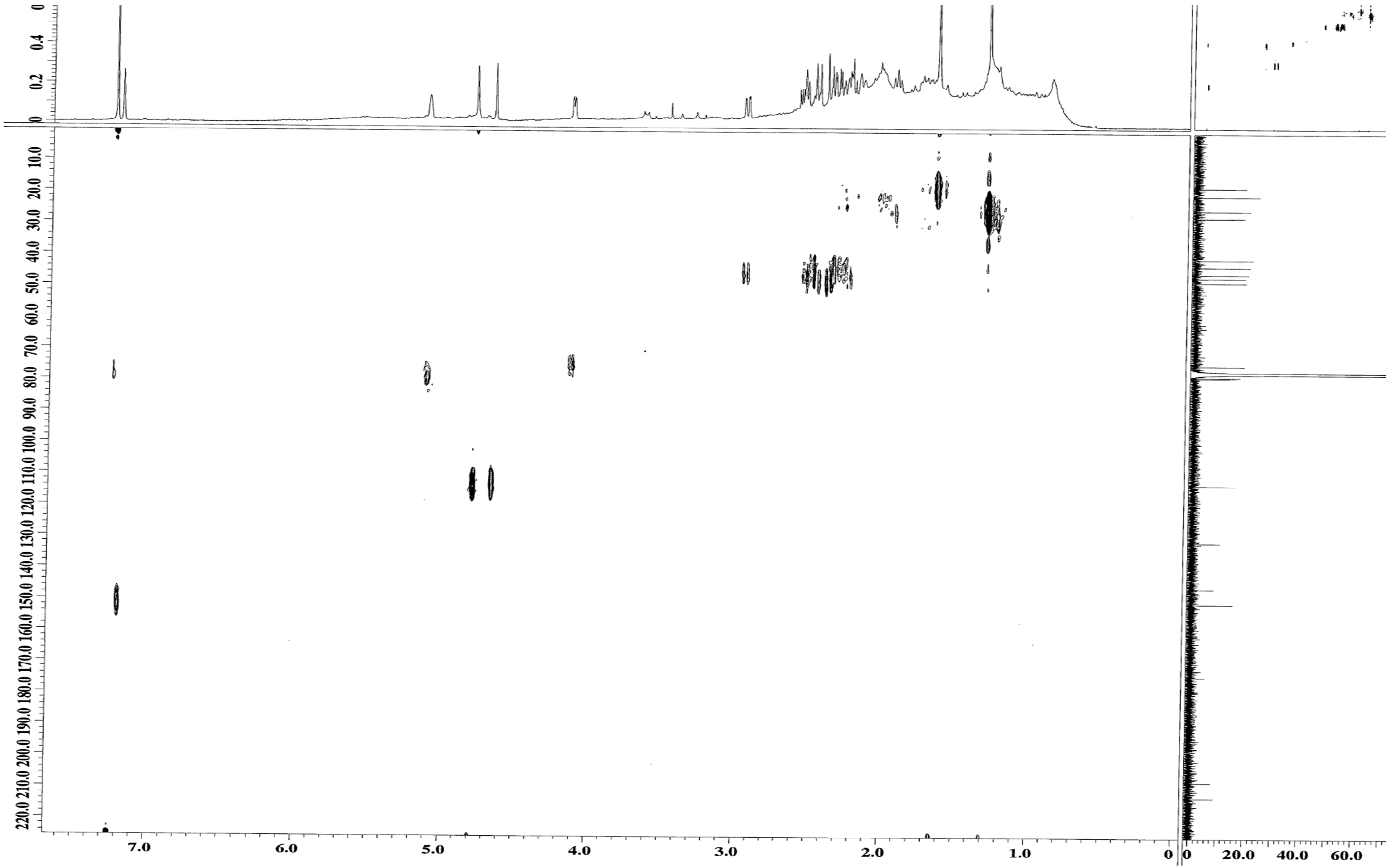

Figure S23. HMQC spectrum of SP-3-13-3 (norcembrenolide B) (**4**) in $CDCl_3$.

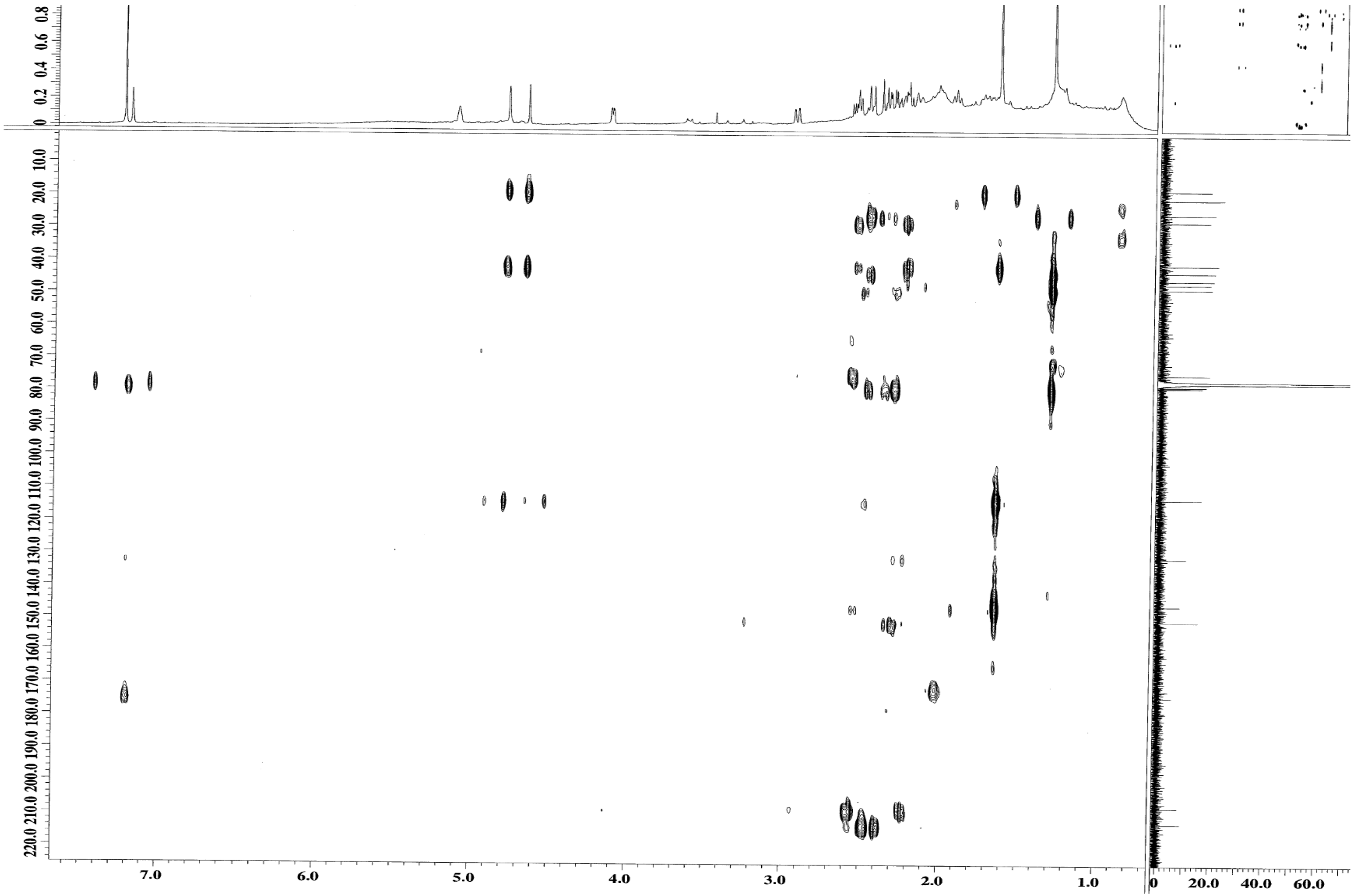

Figure S24. HMBC spectrum of SP-3-13-3 (norcembrenolide B) (**4**) in $CDCl_3$.

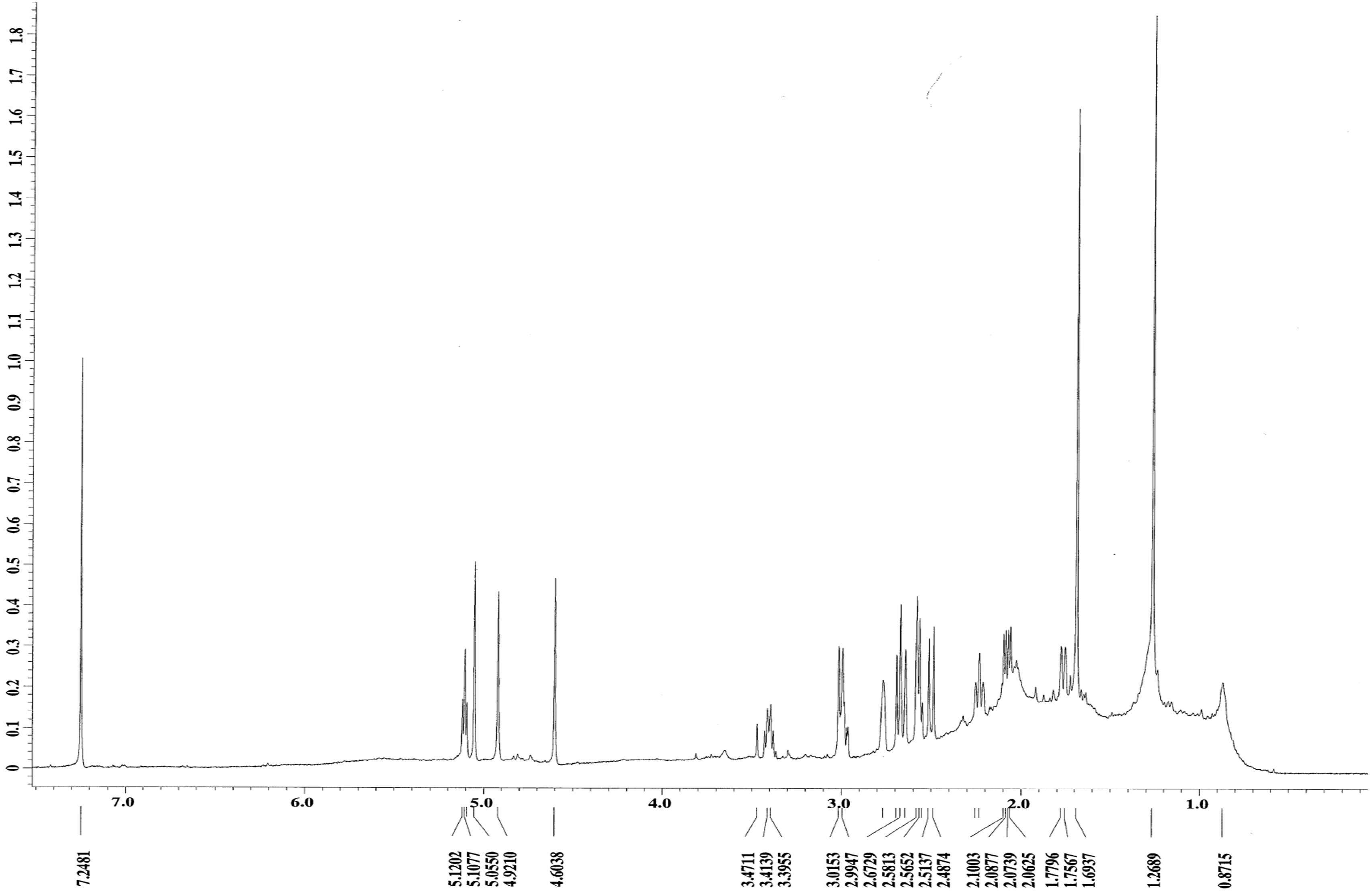

Figure S25. ^{1}H-NMR spectrum of SP-3-13-2 (Ineleganolide) (**5**) in $CDCl_3$.

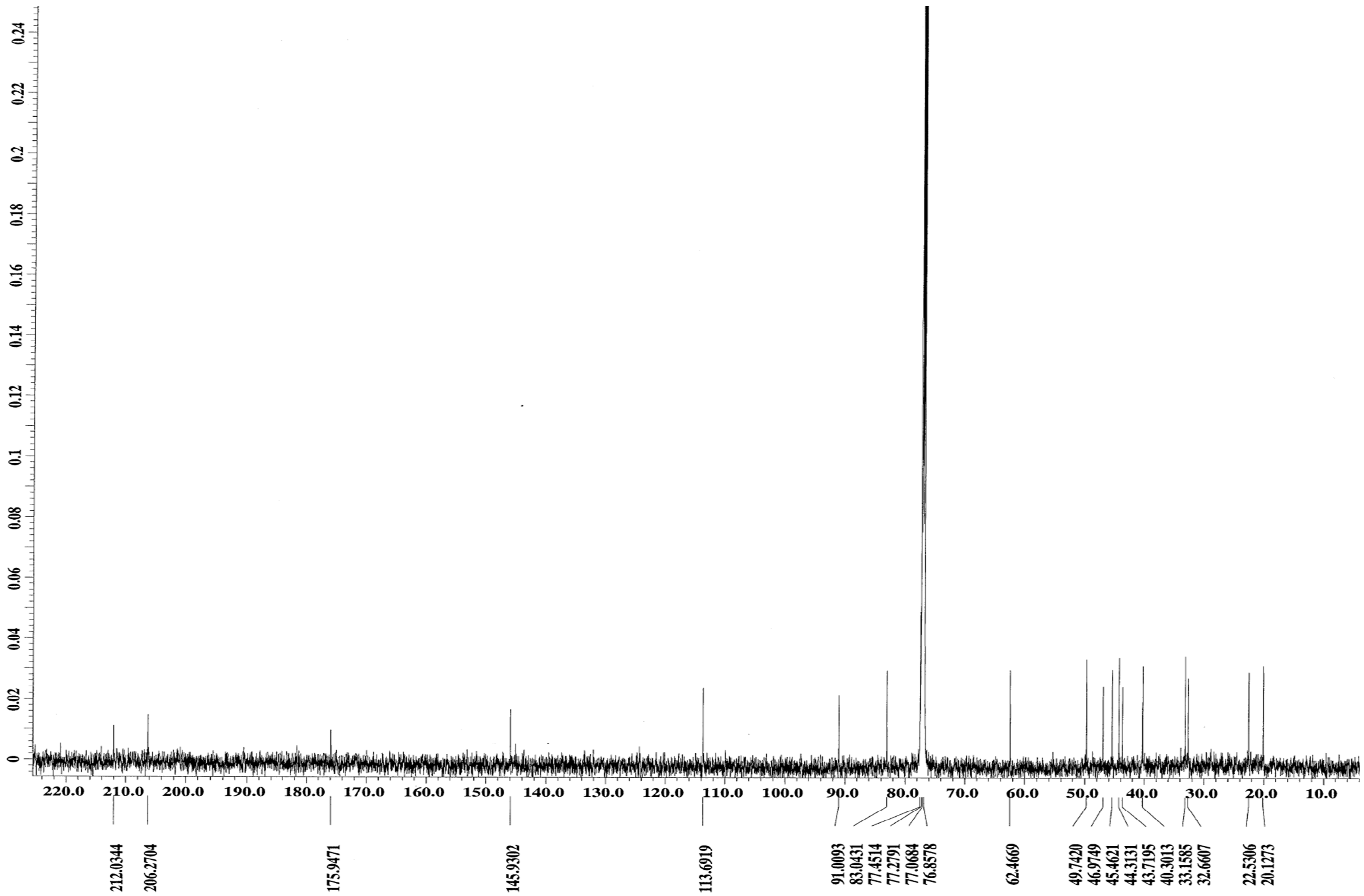

Figure S26. ^{13}C-NMR spectrum of SP-3-13-2 (Ineleganolide) (**5**) in $CDCl_3$.

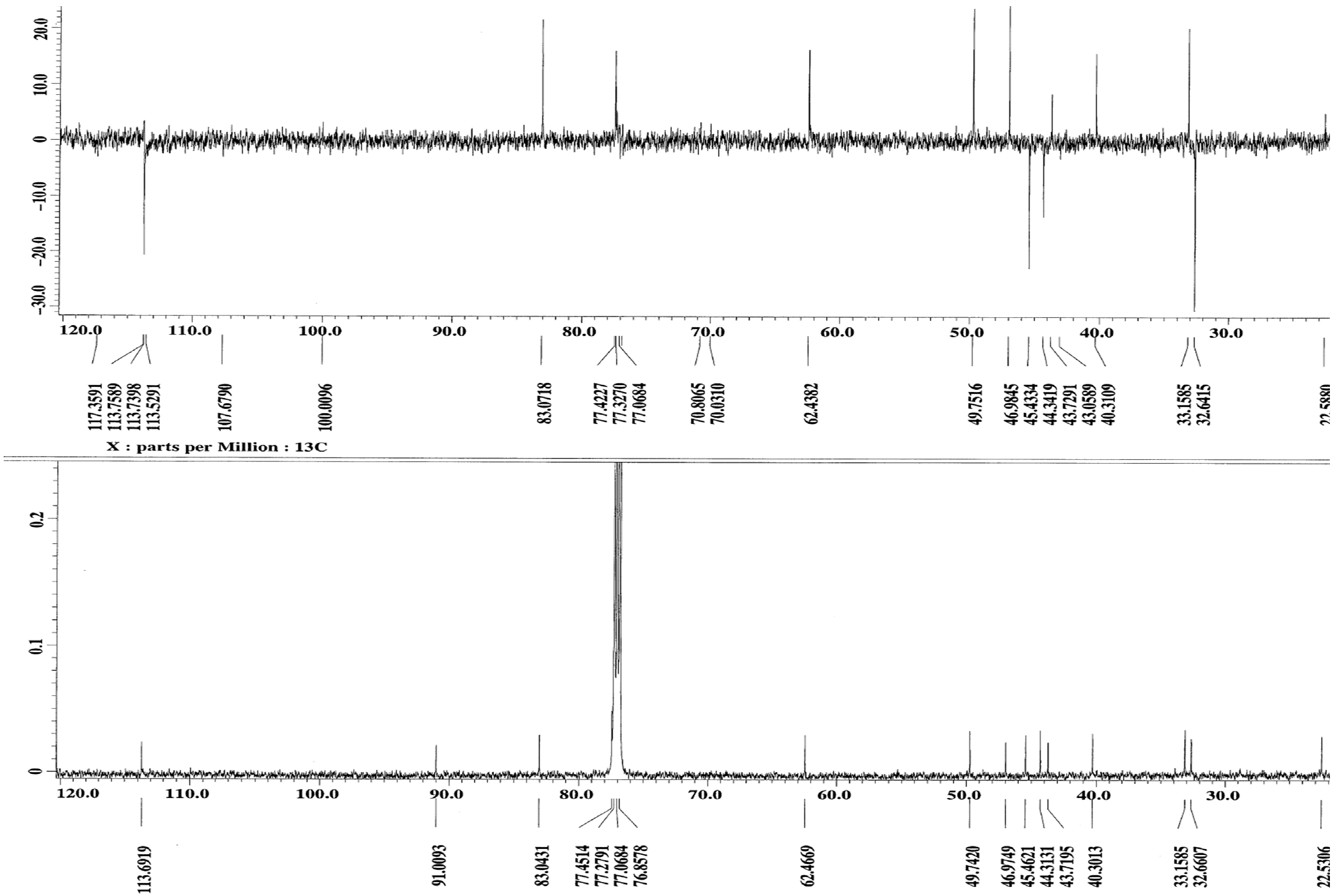

Figure S27. DEPT spectrum of SP-3-13-2 (Ineleganolide) (**5**) in $CDCl_3$.

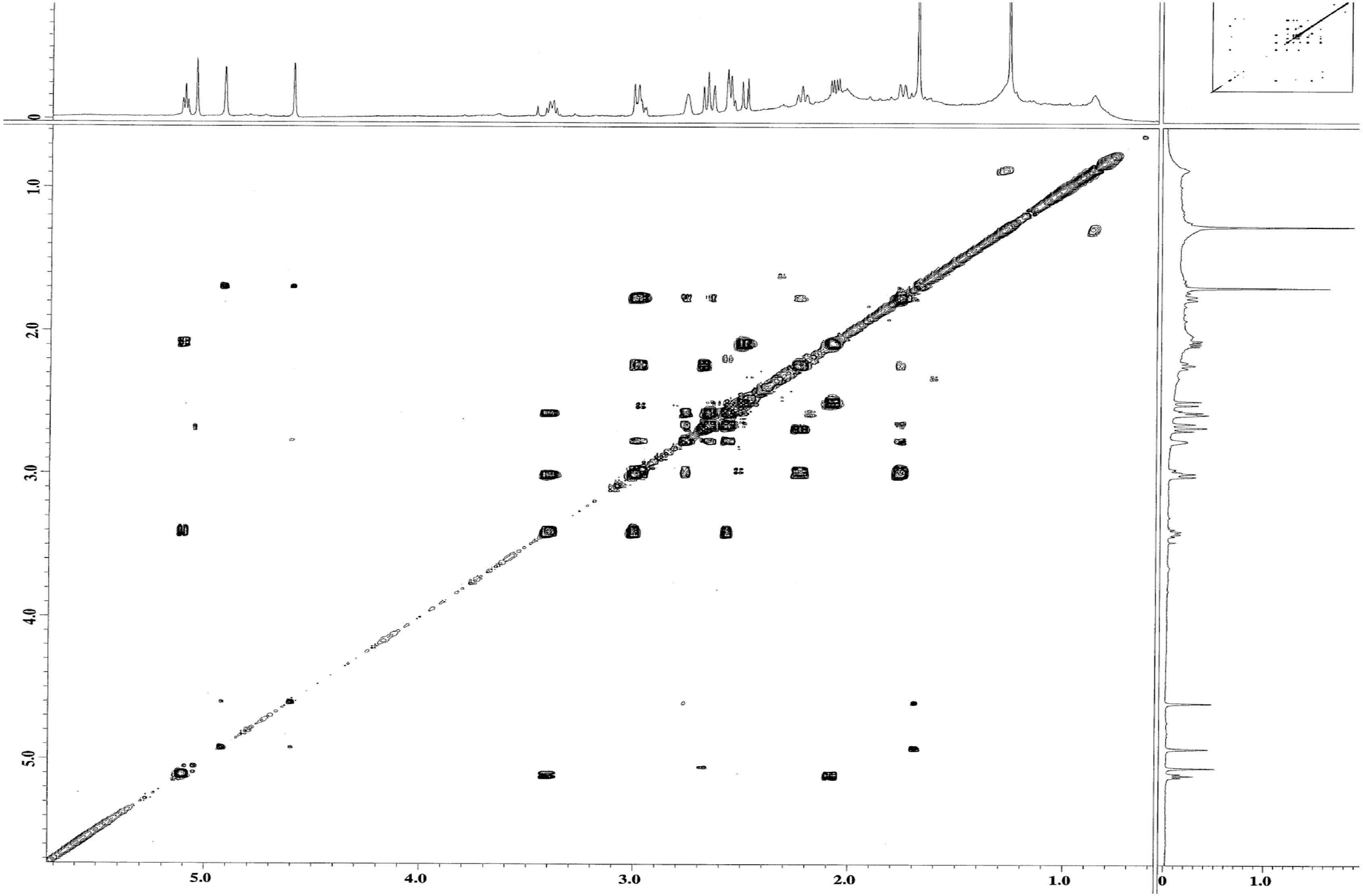

Figure S28. ^{1}H-^{1}H COSY spectrum of SP-3-13-2(Ineleganolide) (**5**) in $CDCl_3$.

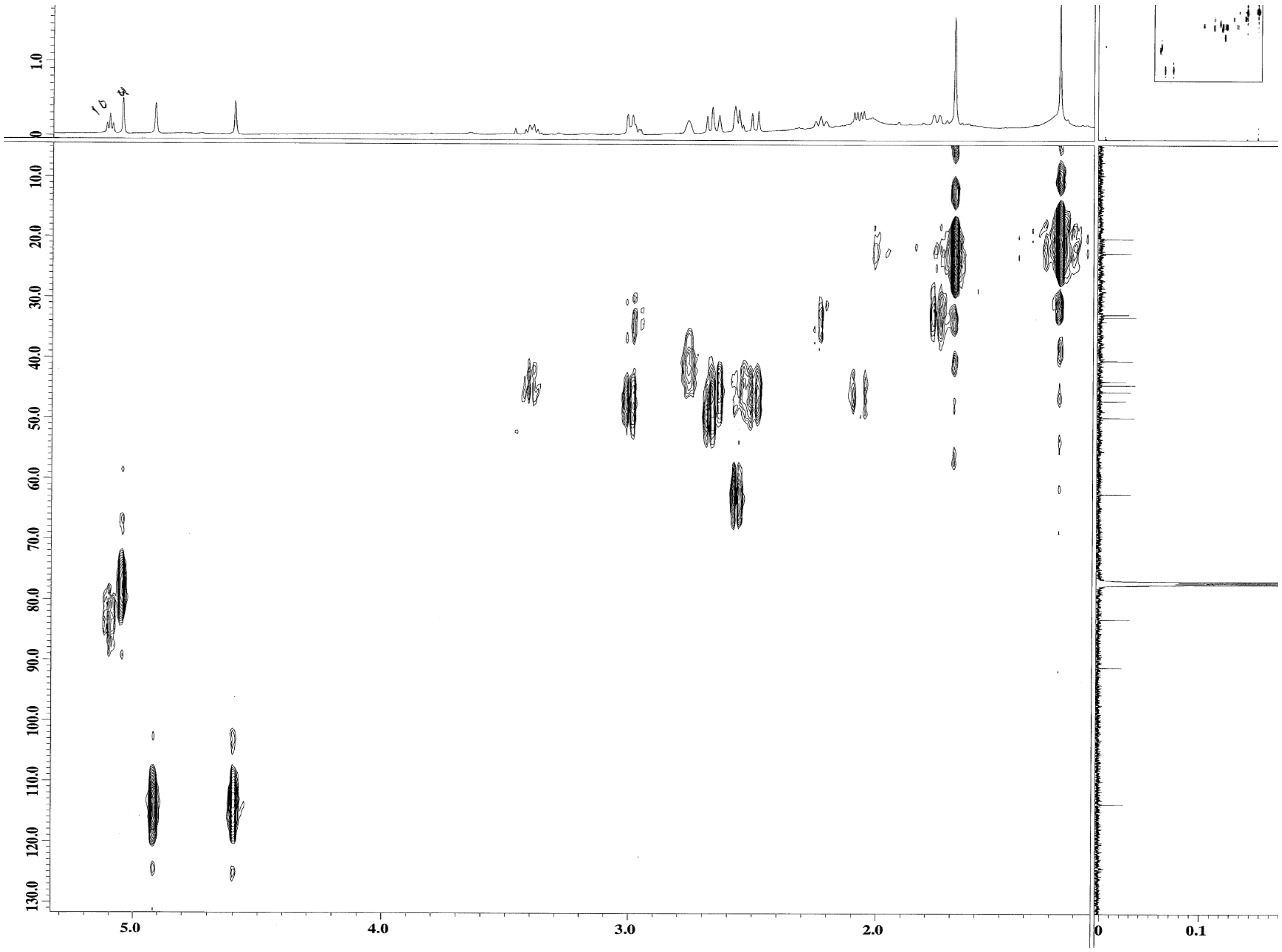

Figure S29. HMQC spectrum of SP-3-13-2(Ineleganolide) (**5**) in $CDCl_3$.

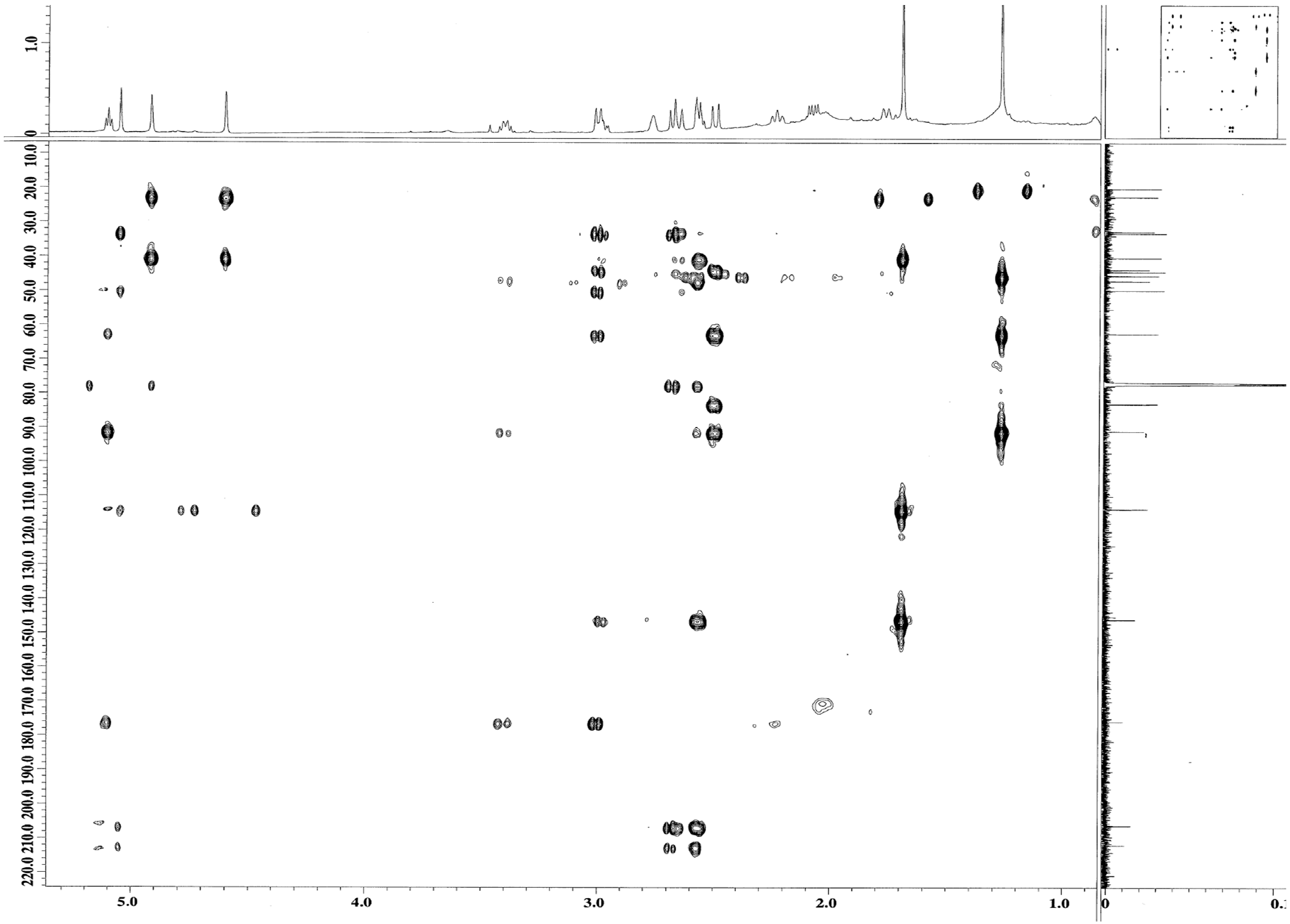

Figure S30. HMBC spectrum of SP-3-13-2(Ineleganolide) (**5**) in $CDCl_3$.

Crystal Structure Report for Compound 2

A specimen of $C_{20}H_{32}O_2$ was used for the X-ray crystallographic analysis. The X-ray intensity data were measured.

Table S1. Data collection details for Compound **2**.

Axis	dx/mm	2θ/°	ω/°	φ/°	χ/°	Width/°	Frames	Time/s	Wavelength/Å	Voltage/kV	Current/mA	Temperature/K
Omega	59.165	−28.00	−28.00	0.00	54.74	0.50	360	2.00	0.71073	50	24.0	n/a
Omega	59.165	−28.00	−28.00	90.00	54.74	0.50	240	2.00	0.71073	50	24.0	n/a
Omega	59.165	−28.00	−28.00	180.00	54.74	0.50	120	2.00	0.71073	50	24.0	n/a

A total of 720 frames were collected. The total exposure time was 0.40 h. The frames were integrated with the Bruker SAINT software package using a narrow-frame algorithm. The integration of the data using a triclinic unit cell yielded a total of 5460 reflections to a maximum θ angle of 27.85° (0.76 Å resolution), of which 4717 were independent (average redundancy 1.158, completeness = 95.8%, R_{int} = 2.69%, R_{sig} = 6.64%) and 3571 (75.70%) were greater than 2σ (F^2). The final cell constants of a = 9.437(4) Å, b = 9.499(4) Å, c = 10.543(4) Å, α = 98.763(6)°, β = 95.390(5)°, γ = 99.422(5)°, volume = 914.6(6) $Å^3$, are based upon the refinement of the XYZ-centroids of 1256 reflections above 20 σ(I) with 5.389° < 2θ < 41.63°. Data were corrected for absorption effects using the multi-scan method (SADABS). The ratio of minimum to maximum apparent transmission was 0.750. The final anisotropic full-matrix least-squares refinement on F^2 with 409 variables converged at R_1 = 7.37%, for the observed data and wR_2 = 21.18% for all data. The goodness-of-fit was 1.020. The largest peak in the final difference electron density synthesis was 0.380 $e^-/Å^3$ and the largest hole was −0.229 $e^-/Å^3$ with an RMS deviation of 0.064 $e^-/Å^3$. On the basis of the final model, the calculated density was 1.071 g/cm^3 and F(000), 325 e^-.

Table S2. Sample and crystal data for Compound **2**.

Identification code	SP4373	
Chemical formula	$C_{20}H_{32}O_2$	
Formula weight	589.88 g/mol	
Temperature	100(2) K	
Wavelength	0.71073 Å	
Crystal system	triclinic	
Space group	P 1	
Unit cell dimensions	a = 9.437(4) Å	α = 98.763(6)°
	b = 9.499(4) Å	β = 95.390(5)°
	c = 10.543(4) Å	γ = 99.422(5)°
Volume	914.6(6) Å3	
Z	1	
Density (calculated)	1.071 g/cm^3	
Absorption coefficient	0.065 mm^{-1}	
F(000)	325	

Table S3. Data collection and structure refinement for Compound **2**.

Theta range for data collection	1.97° to 27.85°
Index ranges	−11 ≤ h ≤ 12, −12 ≤ k ≤ 7, −13 ≤ l ≤ 13
Reflections collected	5460
Independent reflections	4717 (R_{int} = 0.0269)
Coverage of independent reflections	95.8%
Absorption correction	multi-scan
Refinement method	Full-matrix least-squares on F2
Refinement program	SHELXL-2014/6 (Sheldrick, 2014)
Function minimized	Σ w(F_o^2 − F_c^2)^2
Data/restraints/parameters	4717/3/409
Goodness-of-fit on F2	1.020
Δ/σ max	0.012
Final R indices	3571 data; I > 2σ (I) R_1 = 0.0737, wR_2 = 0.1912
	all data R_1 = 0.0926, wR_2 = 0.2118
Weighting scheme	w = 1/[σ^2(F_o^2) + (0.1387P)^2] where P = (F_o^2 + 2F_c^2)/3
Absolute structure parameter	0.9(10)
Largest diff. peak and hole	0.380 and −0.229 eÅ^{-3}
R.M.S. deviation from mean	0.064 eÅ^{-3}

Table S4. Atomic coordinates and equivalent isotropic atomic displacement parameters (Å2) for Compound **2**. U(eq) is defined as one third of the trace of the orthogonalized Uij tensor.

	**x/a**	**y/b**	**z/c**	**U(eq)**
O1	0.3165(5)	0.3469(6)	0.6633(4)	0.0603(13)
O2	0.3133(4)	0.4465(4)	0.9214(3)	0.0378(8)
O3	0.1224(4)	0.1935(4)	0.9034(4)	0.0477(10)
C20	0.3410(8)	0.6095(11)	0.5713(8)	0.073(2)
C12	0.4329(6)	0.5235(7)	0.5430(5)	0.0424(13)
C13	0.5620(6)	0.5667(7)	0.4730(5)	0.0440(13)
C14	0.6869(5)	0.6683(6)	0.5596(5)	0.0351(11)
C1	0.8182(5)	0.6870(6)	0.4866(5)	0.0329(10)
C2	0.9238(5)	0.5819(5)	0.4864(5)	0.0312(10)
C3	0.9125(5)	0.4673(5)	0.5669(4)	0.0288(10)
C4	0.9234(5)	0.3293(5)	0.5312(4)	0.0300(10)
C5	0.9158(5)	0.2281(5)	0.6263(5)	0.0305(10)
O5	0.0510(4)	0.1781(4)	0.6463(3)	0.0397(8)
C11	0.4198(5)	0.3755(7)	0.5758(5)	0.0404(12)
C10	0.3755(6)	0.2551(8)	0.4582(5)	0.0489(15)
C9	0.3964(6)	0.1087(8)	0.4826(6)	0.0537(16)
C8	0.5500(6)	0.0786(7)	0.4771(6)	0.0532(16)
C7	0.6478(6)	0.1127(6)	0.5791(6)	0.0428(13)
C6	0.8014(6)	0.0895(6)	0.5878(6)	0.0444(13)
C15	0.9718(5)	0.7410(5)	0.5443(5)	0.0313(10)
C17	0.0666(6)	0.8308(6)	0.4655(5)	0.0380(12)
C16	0.0097(6)	0.7877(5)	0.6868(5)	0.0359(11)
C18	0.9469(7)	0.2669(6)	0.3961(5)	0.0469(14)
C37	0.6568(5)	0.4433(6)	0.1230(5)	0.0352(11)
C35	0.7476(5)	0.5343(5)	0.0433(4)	0.0294(10)
C22	0.9064(5)	0.5831(5)	0.1000(4)	0.0275(9)
C23	0.0223(5)	0.5886(5)	0.0162(4)	0.0264(9)
C24	0.1605(5)	0.5762(5)	0.0474(4)	0.0277(9)
C25	0.2632(5)	0.5805(5)	0.9462(5)	0.0304(10)
C26	0.3980(5)	0.6994(6)	0.9853(6)	0.0402(12)
C27	0.3695(6)	0.8468(6)	0.0194(6)	0.0444(13)
C28	0.4073(6)	0.9635(7)	0.9668(7)	0.0565(18)

Table S4. *Cont.*

	x/a	y/b	z/c	U(eq)
C39	0.4880(9)	0.9582(11)	0.8491(9)	0.085(3)
C36	0.7067(5)	0.4887(6)	0.8998(5)	0.0353(11)
C29	0.3837(7)	0.1104(7)	0.0219(9)	0.069(2)
C30	0.2433(7)	0.1221(6)	0.0822(6)	0.0518(16)
C31	0.1111(6)	0.0833(5)	0.9818(5)	0.0348(11)
C32	0.9712(6)	0.0686(5)	0.0414(5)	0.0362(11)
C40	0.8867(8)	0.1621(8)	0.0379(7)	0.0615(18)
C33	0.9375(6)	0.9376(6)	0.1086(5)	0.0404(12)
C34	0.8231(6)	0.8160(6)	0.0294(5)	0.0349(11)
C21	0.8013(5)	0.6899(5)	0.1028(5)	0.0292(10)
C38	0.2198(6)	0.5544(6)	0.1799(5)	0.0396(12)
C19	0.5774(9)	0.0087(13)	0.3474(8)	0.105(4)

Table S5. Bond lengths (Å) for Compound **2**.

Atom	Distance	Atom	Distance
O1-C11	1.427(6)	O1-H11A	0.84
O2-C25	1.425(6)	O2-H25A	0.84
O3-C31	1.426(6)	O3-H31A	0.84
C20-12	1.309(9)	C20-H29	0.95
C20-28	0.95	C12-C11	1.487(9)
C12-C13	1.516(7)	C13-C14	1.520(8)
C13-H12	0.99	C13-H13	0.99
C14-C1	1.520(7)	C14-H14	0.99
C14-H15	0.99	C1-C15	1.494(7)
C1-C2	1.521(7)	C1-H1	1.0
C2-C3	1.475(6)	C2-C15	1.518(7)
C2-H2	1.0	C3-C4	1.331(7)
C3-H3	0.95	C4-C5	1.490(6)
C4-C18	1.508(7)	C5-O5	1.441(6)
C5-C6	1.534(7)	C5-H4	1.0
O5-H5A	0.84	C11-C10	1.528(9)
C11-H11	1.0	C10-C9	1.492(10)
C10-H10	0.99	C10-H9	0.99
C9-C8	1.527(9)	C9-H64	0.99
C9-H8	0.99	C8-C7	1.313(8)
C8-C19	1.491(10)	C7-C6	1.498(8)
C7-H7	0.95	C6-H5	0.99
C6-H6	0.99	C15-C16	1.491(7)
C15-C17	1.524(6)	C17-H21	0.98
C17-H20	0.98	C17-H19	0.98
C16-H16	0.98	C16-H17	0.98
C16-H18	0.98	C18-H18A	0.98
C18-H18B	0.98	C18-H18C	0.98
C37-C35	1.517(6)	C37-H51	0.98
C37-H52	0.98	C37-H50	0.98
C35-C21	1.499(7)	C35-C36	1.504(7)
C35-C22	1.529(6)	C22-C23	1.469(6)
C22-C21	1.529(7)	C22-H32	1.0
C23-C24	1.343(6)	C23-H33	0.95
C24-C25	1.508(6)	C24-C38	1.512(6)
C25-C26	1.534(7)	C25-H34	1.0
C26-C27	1.466(8)	C26-H35	0.99
C26-H36	0.99	C27-C28	1.327(7)
C27-H37	0.95	C28-C29	1.487(11)
C28-C39	1.514(11)	C39-H56	0.98

Table S5. *Cont.*

Atom	Distance	Atom	Distance
C39-H58	0.98	C39-H57	0.98
C36-H47	0.98	C36-H49	0.98
C36-H48	0.98	C29-C30	1.535(11)
C29-H39	0.99	C29-H38	0.99
C30-C31	1.515(7)	C30-H40	0.99
C30-H41	0.99	C31-C32	1.509(8)
C31-H42	1.0	C32-C40	1.290(8)
C32-C33	1.527(6)	C40-H59	0.95
C40-H60	0.95	C33-C34	1.525(8)
C33-H44	0.99	C33-H43	0.99
C34-C21	1.519(7)	C34-H46	0.99
C34-H45	0.99	C21-H31	1.0
C38-H55	0.98	C38-H54	0.98
C38-H53	0.98	C19-H19A	0.98
C19-H19B	0.98	C19-H19C	0.98

Table S6. Bond angles (°) for Compound **2**.

Atom	Angle	Atom	Angle
C11-O1-H11A	109.5	C25-O2-H25A	109.5
C31-O3-H31A	109.5	C12-C20-H29	120
C12-C20-H28	120	H29-C20-H28	120
C20-C12-C11	123.3(6)	C20-C12-C13	122.6(6)
C11-C12-C13	114.1(5)	C12-C13-C14	113.4(4)
C12-C13-H12	108.9	C14-C13-H12	108.9
C12-C13-H13	108.9	C14-C13-H13	108.9
H12-C13-H13	107.7	C1-C14-C13	109.3(4)
C1-C14-H14	109.8	C13-C14-H14	109.8
C1-C14-H15	109.8	C13-C14-H15	109.8
H14-C14-H15	108.3	C15-C1-C14	126.5(4)
C15-C1-C2	60.5(3)	C14-C1-C2	120.9(4)
C15-C1-H1	113	C14-C1-H1	113
C2-C1-H1	113	C3-C2-C15	122.2(4)
C3-C2-C1	122.0(4)	C15-C2-C1	58.9(3)
C3-C2-H2	114.3	C15-C2-H2	114.3
C1-C2-H2	114.3	C4-C3-C2	127.2(4)
C4-C3-H3	116.4	C2-C3-H3	116.4
C3-C4-C5	120.6(4)	C3-C4-C18	123.1(4)
C5-C4-C18	116.3(4)	O5-C5-C4	110.6(4)
O5-C5-C6	104.7(4)	C4-C5-C6	115.7(4)
O5-C5-H4	108.5	C4-C5-H4	108.5
C6-C5-H4	108.5	C5-O5-H5A	109.5
O1-C11-C12	113.5(5)	O1-C11-C10	105.8(4)
C12-C11-C10	113.5(4)	O1-C11-H11	107.9
C12-C11-H11	107.9	C10-C11-H11	107.9
C9-C10-C11	114.5(5)	C9-C10-H10	108.6
C11-C10-H10	108.6	C9-C10-H9	108.6
C11-C10-H9	108.6	H10-C10-H9	107.6
C10-C9-C8	115.3(5)	C10-C9-H64	108.5
C8-C9-H64	108.5	C10-C9-H8	108.5
C8-C9-H8	108.5	H64-C9-H8	107.5
C7-C8-C19	123.9(6)	C7-C8-C9	121.8(5)
C19-C8-C9	114.3(6)	C8-C7-C6	127.5(6)
C8-C7-H7	116.2	C6-C7-H7	116.2
C7-C6-C5	114.9(5)	C7-C6-H5	108.5
C5-C6-H5	108.5	C7-C6-H6	108.5

Table S6. *Cont.*

Atom	Angle	Atom	Angle
C5-C6-H6	108.5	H5-C6-H6	107.5
C16-C15-C1	120.8(4)	C16-C15-C2	120.7(4)
C1-C15-C2	60.7(3)	C16-C15-C17	113.4(4)
C1-C15-C17	116.0(4)	C2-C15-C17	115.5(4)
C15-C17-H21	109.5	C15-C17-H20	109.5
H21-C17-H20	109.5	C15-C17-H19	109.5
H21-C17-H19	109.5	H20-C17-H19	109.5
C15-C16-H16	109.5	C15-C16-H17	109.5
H16-C16-H17	109.5	C15-C16-H18	109.5
H16-C16-H18	109.5	H17-C16-H18	109.5
C4-C18-H18A	109.5	C4-C18-H18B	109.5
H18A-C18-H18B	109.5	C4-C18-H18C	109.5
H18A-C18-H18C	109.5	H18B-C18-H18C	109.5
C35-C37-H51	109.5	C35-C37-H52	109.5
H51-C37-H52	109.5	C35-C37-H50	109.5
H51-C37-H50	109.5	H52-C37-H50	109.5
C21-C35-C36	121.5(4)	C21-C35-C37	116.2(4)
C36-C35-C37	113.7(4)	C21-C35-C22	60.7(3)
C36-C35-C22	120.5(4)	C37-C35-C22	114.4(4)
C23-C22-C35	121.1(4)	C23-C22-C21	121.7(4)
C35-C22-C21	58.7(3)	C23-C22-H32	114.7
C35-C22-H32	114.7	C21-C22-H32	114.7
C24-C23-C22	127.3(4)	C24-C23-H33	116.4
C22-C23-H33	116.4	C23-C24-C25	119.5(4)
C23-C24-C38	123.3(4)	C25-C24-C38	117.1(4)
O2-C25-C24	110.4(4)	O2-C25-C26	106.7(4)
C24-C25-C26	113.5(4)	O2-C25-H34	108.7
C24-C25-H34	108.7	C26-C25-H34	108.7
C27-C26-C25	115.3(4)	C27-C26-H35	108.4
C25-C26-H35	108.4	C27-C26-H36	108.4
C25-C26-H36	108.4	H35-C26-H36	107.5
C28-C27-C26	130.2(6)	C28-C27-H37	114.9
C26-C27-H37	114.9	C27-C28-C29	123.6(7)
C27-C28-C39	121.7(7)	C29-C28-C39	114.6(6)
C28-C39-H56	109.5	C28-C39-H58	109.5
H56-C39-H58	109.5	C28-C39-H57	109.5
H56-C39-H57	109.5	H58-C39-H57	109.5
C35-C36-H47	109.5	C35-C36-H49	109.5
H47-C36-H49	109.5	C35-C36-H48	109.5
H47-C36-H48	109.5	H49-C36-H48	109.5
C28-C29-C30	117.9(5)	C28-C29-H39	107.8
C30-C29-H39	107.8	C28-C29-H38	107.8
C30-C29-H38	107.8	H39-C29-H38	107.2
C31-C30-C29	112.2(5)	C31-C30-H40	109.2
C29-C30-H40	109.2	C31-C30-H41	109.2
C29-C30-H41	109.2	H40-C30-H41	107.9
O3-C31-C32	112.0(4)	O3-C31-C30	106.5(4)
C32-C31-C30	112.6(4)	O3-C31-H42	108.6
C32-C31-H42	108.6	C30-C31-H42	108.6
C40-C32-C31	122.1(5)	C40-C32-C33	122.0(5)
C31-C32-C33	115.8(4)	C32-C40-H59	120
C32-C40-H60	120	H59-C40-H60	120
C34-C33-C32	113.3(4)	C34-C33-H44	108.9
C32-C33-H44	108.9	C34-C33-H43	108.9
C32-C33-H43	108.9	H44-C33-H43	107.7

Table S6. *Cont.*

Atom	Angle	Atom	Angle
C21-C34-C33	109.0(4)	C21-C34-H46	109.9
C33-C34-H46	109.9	C21-C34-H45	109.9
C33-C34-H45	109.9	H46-C34-H45	108.3
C35-C21-C34	125.6(4)	C35-C21-C22	60.6(3)
C34-C21-C22	120.8(4)	C35-C21-H31	113.3
C34-C21-H31	113.3	C22-C21-H31	113.3
C24-C38-H55	109.5	C24-C38-H54	109.5
H55-C38-H54	109.5	C24-C38-H53	109.5
H55-C38-H53	109.5	H54-C38-H53	109.5
C8-C19-H19A	109.5	C8-C19-H19B	109.5
H19A-C19-H19B	109.5	C8-C19-H19C	109.5
H19A-C19-H19C	109.5	H19B-C19-H19C	109.5

Table S7. Torsion angles (°) for Compound **2**.

Atom	Angle	Atom	Angle
C20-C12-C13-C14	77.7(8)	C11-C12-C13-C14	−102.5(6)
C12-C13-C14-C1	171.6(5)	C13-C14-C1-C15	−161.3(5)
C13-C14-C1-C2	−87.1(5)	C15-C1-C2-C3	110.9(5)
C14-C1-C2-C3	−6.4(7)	C14-C1-C2-C15	−117.2(5)
C15-C2-C3-C4	−153.9(5)	C1-C2-C3-C4	135.2(5)
C2-C3-C4-C5	177.3(4)	C2-C3-C4-C18	−1.4(8)
C3-C4-C5-O5	−115.7(5)	C18-C4-C5-O5	63.0(6)
C3-C4-C5-C6	125.4(5)	C18-C4-C5-C6	−55.9(6)
C20-C12-C11-O1	−12.1(8)	C13-C12-C11-O1	168.1(5)
C20-C12-C11-C10	108.8(7)	C13-C12-C11-C10	−71.0(6)
O1-C11-C10-C9	−69.2(6)	C12-C11-C10-C9	165.7(5)
C11-C10-C9-C8	−82.0(6)	C10-C9-C8-C7	87.7(7)
C10-C9-C8-C19	−92.1(8)	C19-C8-C7-C6	−0.3(11)
C9-C8-C7-C6	179.9(6)	C8-C7-C6-C5	119.2(7)
O5-C5-C6-C7	172.0(4)	C4-C5-C6-C7	−65.9(6)
C14-C1-C15-C16	−2.0(8)	C2-C1-C15-C16	−110.3(5)
C14-C1-C15-C2	108.3(5)	C14-C1-C15-C17	−145.6(5)
C2-C1-C15-C17	106.0(5)	C3-C2-C15-C16	−0.1(7)
C1-C2-C15-C16	110.5(5)	C3-C2-C15-C1	−110.5(5)
C3-C2-C15-C17	142.7(5)	C1-C2-C15-C17	−106.8(5)
C21-C35-C22-C23	−110.6(5)	C36-C35-C22-C23	0.9(7)
C37-C35-C22-C23	141.9(4)	C36-C35-C22-C21	111.4(5)
C37-C35-C22-C21	−107.5(5)	C35-C22-C23-C24	−153.5(5)
C21-C22-C23-C24	136.4(5)	C22-C23-C24-C25	178.3(4)
C22-C23-C24-C38	−0.3(8)	C23-C24-C25-O2	−117.0(5)
C38-C24-C25-O2	61.7(5)	C23-C24-C25-C26	123.2(5)
C38-C24-C25-C26	−58.1(6)	O2-C25-C26-C27	−178.0(4)
C24-C25-C26-C27	−56.1(6)	C25-C26-C27-C28	−119.8(6)
C26-C27-C28-C29	−173.1(5)	C26-C27-C28-C39	2.8(10)
C27-C28-C29-C30	−37.8(9)	C39-C28-C29-C30	146.0(6)
C28-C29-C30-C31	−68.6(7)	C29-C30-C31-O3	−67.1(5)
C29-C30-C31-C32	169.8(4)	O3-C31-C32-C40	−10.9(8)
C30-C31-C32-C40	109.0(7)	O3-C31-C32-C33	170.3(4)
C30-C31-C32-C33	−69.7(6)	C40-C32-C33-C34	77.8(7)
C31-C32-C33-C34	−103.5(5)	C32-C33-C34-C21	178.2(4)
C36-C35-C21-C34	−1.1(7)	C37-C35-C21-C34	−146.8(5)
C22-C35-C21-C34	108.7(5)	C36-C35-C21-C22	−109.7(5)
C37-C35-C21-C22	104.5(4)	C33-C34-C21-C35	−154.5(5)
C33-C34-C21-C22	−80.5(5)	C23-C22-C21-C35	109.6(5)
C23-C22-C21-C34	−6.6(7)	C35-C22-C21-C34	−116.2(5)

Table S8. Anisotropic atomic displacement parameters (Å^2) for Compound **2**.

	U^{11}	U^{22}	U^{33}	U^{23}	U^{13}	U^{12}
O1	0.047(2)	0.091(4)	0.034(2)	0.008(2)	0.0172(18)	−0.017(2)
O2	0.0324(18)	0.044(2)	0.0382(19)	0.0077(15)	0.0058(15)	0.0082(15)
O3	0.051(2)	0.049(2)	0.044(2)	0.0231(18)	0.0044(18)	−0.0001(19)
C20	0.051(4)	0.099(6)	0.089(6)	0.040(5)	0.030(4)	0.036(4)
C12	0.030(3)	0.064(4)	0.036(3)	0.015(2)	0.004(2)	0.010(2)
C13	0.034(3)	0.062(4)	0.038(3)	0.014(3)	0.011(2)	0.005(3)
C14	0.033(3)	0.043(3)	0.032(3)	0.012(2)	0.005(2)	0.009(2)
C1	0.034(3)	0.039(3)	0.026(2)	0.011(2)	0.0057(19)	0.004(2)
C2	0.037(3)	0.029(2)	0.027(2)	0.0058(19)	0.0087(19)	0.000(2)
C3	0.027(2)	0.032(2)	0.025(2)	0.0066(18)	0.0048(18)	−0.0011(18)
C4	0.023(2)	0.036(3)	0.031(2)	0.0111(19)	0.0018(17)	−0.0018(18)
C5	0.030(2)	0.028(2)	0.033(2)	0.0060(18)	0.0033(19)	0.0027(19)
O5	0.039(2)	0.040(2)	0.042(2)	0.0108(17)	−0.0006(15)	0.0117(16)
C11	0.021(2)	0.065(4)	0.033(3)	0.011(2)	0.0052(19)	−0.002(2)
C10	0.026(3)	0.081(5)	0.035(3)	0.011(3)	0.006(2)	−0.005(3)
C9	0.033(3)	0.070(4)	0.048(3)	0.005(3)	0.004(2)	−0.014(3)
C8	0.037(3)	0.058(4)	0.055(4)	−0.005(3)	0.011(3)	−0.008(3)
C7	0.037(3)	0.036(3)	0.053(3)	0.012(2)	0.009(2)	−0.008(2)
C6	0.037(3)	0.033(3)	0.063(4)	0.011(2)	0.004(3)	0.002(2)
C15	0.033(2)	0.028(2)	0.035(3)	0.012(2)	0.010(2)	0.0038(19)
C17	0.040(3)	0.034(3)	0.041(3)	0.016(2)	0.008(2)	0.001(2)
C16	0.041(3)	0.026(2)	0.037(3)	0.005(2)	−0.001(2)	0.002(2)
C18	0.064(4)	0.044(3)	0.032(3)	0.005(2)	0.001(2)	0.013(3)
C37	0.024(2)	0.040(3)	0.044(3)	0.014(2)	0.012(2)	0.003(2)
C35	0.024(2)	0.036(3)	0.028(2)	0.0074(19)	0.0053(18)	0.0017(19)
C22	0.029(2)	0.025(2)	0.026(2)	0.0046(18)	0.0001(17)	−0.0006(18)
C23	0.026(2)	0.031(2)	0.021(2)	0.0070(17)	0.0012(17)	−0.0015(18)
C24	0.029(2)	0.025(2)	0.028(2)	0.0073(17)	0.0037(17)	−0.0004(17)
C25	0.024(2)	0.033(2)	0.034(2)	0.0136(19)	0.0013(18)	−0.0023(18)
C26	0.022(2)	0.041(3)	0.059(3)	0.020(3)	0.003(2)	−0.003(2)
C27	0.031(3)	0.034(3)	0.069(4)	0.021(3)	0.001(2)	−0.002(2)
C28	0.028(3)	0.054(4)	0.091(5)	0.044(4)	−0.008(3)	−0.003(2)
C39	0.073(5)	0.092(6)	0.102(6)	0.061(5)	0.014(5)	0.001(5)
C36	0.024(2)	0.045(3)	0.038(3)	0.008(2)	0.0042(19)	0.005(2)
C29	0.038(3)	0.038(3)	0.125(6)	0.035(4)	−0.020(4)	−0.012(3)
C30	0.067(4)	0.022(2)	0.057(4)	0.009(2)	−0.024(3)	−0.004(3)
C31	0.044(3)	0.024(2)	0.035(3)	0.0068(19)	0.002(2)	0.002(2)
C32	0.055(3)	0.023(2)	0.032(3)	0.0086(19)	0.005(2)	0.007(2)
C40	0.080(5)	0.047(4)	0.070(4)	0.022(3)	0.032(4)	0.023(3)
C33	0.053(3)	0.032(3)	0.038(3)	0.013(2)	0.007(2)	0.006(2)
C34	0.042(3)	0.039(3)	0.026(2)	0.008(2)	0.006(2)	0.009(2)
C21	0.030(2)	0.031(2)	0.029(2)	0.0081(19)	0.0102(18)	0.0027(19)
C38	0.038(3)	0.051(3)	0.032(3)	0.014(2)	0.004(2)	0.011(2)
C19	0.056(5)	0.155(10)	0.073(5)	−0.052(6)	0.000(4)	0.000(5)

The anisotropic atomic displacement factor exponent takes the form: $-2\pi^2[h^2a^{*2}U^{11} + ... + 2hka^*b^*U^{12}]$

Table S9. Hydrogen atomic coordinates and isotropic atomic displacement parameters (Å^2) for Compound **2**.

	x/a	y/b	z/c	U(eq)
H11A	0.3381	0.4089	0.7312	0.09
H25A	0.3146	0.4228	−0.1585	0.057
H31A	0.0877	1.1571	−0.1731	0.072
H29	0.2611	0.5783	0.6149	0.088
H28	0.3543	0.7032	0.5481	0.088
H12	0.531	0.6144	0.4006	0.053
H13	0.5964	0.4782	0.4356	0.053
H14	0.659	0.7635	0.5862	0.042

Table S9. *Cont.*

	x/a	y/b	z/c	U(eq)
H15	0.7105	0.6277	0.6385	0.042
H1	0.7963	0.7144	0.3999	0.039
H2	0.9569	0.5534	0.4004	0.037
H3	0.8956	0.4951	0.6538	0.035
H4	0.8966	0.2812	0.7106	0.037
H5A	1.0748	0.1828	0.7258	0.06
H11	0.5164	0.3654	0.6175	0.048
H10	0.2722	0.2504	0.4275	0.059
H9	0.4323	0.2806	0.3881	0.059
H64	0.3299	0.0349	0.418	0.064
H8	0.3683	0.0969	0.569	0.064
H7	0.6177	0.1572	0.6564	0.051
H5	0.8182	0.0387	0.5028	0.053
H6	0.8151	0.0251	0.6517	0.053
H21	1.0393	0.7923	0.3732	0.057
H20	1.1684	0.826	0.4895	0.057
H19	1.0531	0.9317	0.4831	0.057
H16	1.0155	0.8927	0.7087	0.054
H17	1.1034	0.7627	0.7137	0.054
H18	0.9351	0.7384	0.7316	0.054
H18A	0.8546	0.2457	0.3398	0.07
H18B	0.9856	0.1774	0.3977	0.07
H18C	1.0159	0.337	0.363	0.07
H51	−0.4437	0.4577	0.1102	0.053
H52	−0.3053	0.4726	0.2147	0.053
H50	−0.3394	0.3409	0.0955	0.053
H32	−0.0682	0.5533	0.185	0.033
H33	−0.0033	0.6024	−0.0698	0.032
H34	0.2106	0.5969	−0.1356	0.037
H35	0.4559	0.6764	0.0601	0.048
H36	0.4576	0.6973	−0.0872	0.048
H37	0.3154	0.8609	0.0904	0.053
H56	0.504	0.8593	−0.1781	0.128
H58	0.5814	1.0242	−0.1294	0.128
H57	0.4307	0.9877	−0.2214	0.128
H47	−0.2723	0.3916	−0.1268	0.053
H49	−0.2374	0.5573	−0.1454	0.053
H48	−0.3968	0.4875	−0.1218	0.053
H39	0.3875	1.1695	−0.0477	0.082
H38	0.4659	1.1549	0.089	0.082
H40	0.2328	1.0566	0.1466	0.062
H41	0.2494	1.2224	0.128	0.062
H42	0.1147	0.989	−0.0736	0.042
H59	−0.0883	1.2435	−0.0031	0.074
H60	−0.2006	1.1496	0.0764	0.074
H44	0.0277	0.8997	0.1266	0.048
H43	−0.0964	0.9692	0.1926	0.048
H46	−0.2692	0.851	0.0141	0.042
H45	−0.1451	0.7842	−0.0555	0.042
H31	−0.2296	0.7164	0.1901	0.035
H55	0.2487	0.6486	0.2368	0.059
H54	0.304	0.5069	0.1726	0.059
H53	0.145	0.4936	0.216	0.059
H19A	0.6763	−0.0112	0.3525	0.157
H19B	0.5656	0.0738	0.2849	0.157
H19C	0.5084	−0.0823	0.3194	0.157

Table S10. Hydrogen bond distances (Å) and angles (°) for Compound **2**.

	Donor-H	Acceptor-H	Donor-Acceptor	Angle
O5-H5A...O3	0.84	1.87	2.707(5)	178.4
O3-H31A...O5	0.84	1.95	2.707(5)	149.5
O2-H25A...O1	0.84	1.91	2.745(5)	173.6
O1-H11A...O2	0.84	2.02	2.745(5)	143.5
O5-H5A...O3	0.84	1.87	2.707(5)	178.4
O3-H31A...O5	0.84	1.95	2.707(5)	149.5
O2-H25A...O1	0.84	1.91	2.745(5)	173.6
O1-H11A...O2	0.84	2.02	2.745(5)	143.5
O5-H5A...O3	0.84	1.87	2.707(5)	178.4
O3-H31A...O5	0.84	1.95	2.707(5)	149.5
O2-H25A...O1	0.84	1.91	2.745(5)	173.6
O1-H11A...O2	0.84	2.02	2.745(5)	143.5
O5-H5A...O3	0.84	1.87	2.707(5)	178.4
O3-H31A...O5	0.84	1.95	2.707(5)	149.5
O2-H25A...O1	0.84	1.91	2.745(5)	173.6
O1-H11A...O2	0.84	2.02	2.745(5)	143.5
O5-H5A...O3	0.84	1.87	2.707(5)	178.4
O3-H31A...O5	0.84	1.95	2.707(5)	149.5
O2-H25A...O1	0.84	1.91	2.745(5)	173.6
O1-H11A...O2	0.84	2.02	2.745(5)	143.5
O5-H5A...O3	0.84	1.87	2.707(5)	178.4
O3-H31A...O5	0.84	1.95	2.707(5)	149.5
O2-H25A...O1	0.84	1.91	2.745(5)	173.6
O1-H11A...O2	0.84	2.02	2.745(5)	143.5
O5-H5A...O3	0.84	1.87	2.707(5)	178.4
O3-H31A...O5	0.84	1.95	2.707(5)	149.5
O2-H25A...O1	0.84	1.91	2.745(5)	173.6
O1-H11A...O2	0.84	2.02	2.745(5)	143.5
O5-H5A...O3	0.84	1.87	2.707(5)	178.4
O3-H31A...O5	0.84	1.95	2.707(5)	149.5
O2-H25A...O1	0.84	1.91	2.745(5)	173.6
O1-H11A...O2	0.84	2.02	2.745(5)	143.5
O5-H5A...O3	0.84	1.87	2.707(5)	178.4
O3-H31A...O5	0.84	1.95	2.707(5)	149.5
O2-H25A...O1	0.84	1.91	2.745(5)	173.6
O1-H11A...O2	0.84	2.02	2.745(5)	143.5
O5-H5A...O3	0.84	1.87	2.707(5)	178.4
O3-H31A...O5	0.84	1.95	2.707(5)	149.5
O2-H25A...O1	0.84	1.91	2.745(5)	173.6
O1-H11A...O2	0.84	2.02	2.745(5)	143.5
O5-H5A...O3	0.84	1.87	2.707(5)	178.4
O3-H31A...O5	0.84	1.95	2.707(5)	149.5
O2-H25A...O1	0.84	1.91	2.745(5)	173.6
O1-H11A...O2	0.84	2.02	2.745(5)	143.5
O5-H5A...O3	0.84	1.87	2.707(5)	178.4
O3-H31A...O5	0.84	1.95	2.707(5)	149.5
O2-H25A...O1	0.84	1.91	2.745(5)	173.6
O1-H11A...O2	0.84	2.02	2.745(5)	143.5
O5-H5A...O3	0.84	1.87	2.707(5)	178.4
O3-H31A...O5	0.84	1.95	2.707(5)	149.5
O2-H25A...O1	0.84	1.91	2.745(5)	173.6
O1-H11A...O2	0.84	2.02	2.745(5)	143.5
O1-H11A...O2	0.84	2.02	2.745(5)	143.5
O2-H25A...O1	0.84	1.91	2.745(5)	173.6
O3-H31A...O5	0.84	1.95	2.707(5)	149.5
O5-H5A...O3	0.84	1.87	2.707(5)	178.4
O1-H11A...O2	0.84	2.02	2.745(5)	143.5
O2-H25A...O1	0.84	1.91	2.745(5)	173.6
O3-H31A...O5	0.84	1.95	2.707(5)	149.5
O5-H5A...O3	0.84	1.87	2.707(5)	178.4
O1-H11A...O2	0.84	2.02	2.745(5)	143.5
O2-H25A...O1	0.84	1.91	2.745(5)	173.6

Table S10. *Cont.*

	Donor-H	Acceptor-H	Donor-Acceptor	Angle
O3-H31A...O5	0.84	1.95	2.707(5)	149.5
O5-H5A...O3	0.84	1.87	2.707(5)	178.4
O1-H11A...O2	0.84	2.02	2.745(5)	143.5
O2-H25A...O1	0.84	1.91	2.745(5)	173.6
O3-H31A...O5	0.84	1.95	2.707(5)	149.5
O5-H5A...O3	0.84	1.87	2.707(5)	178.4
O1-H11A...O2	0.84	2.02	2.745(5)	143.5
O2-H25A...O1	0.84	1.91	2.745(5)	173.6
O3-H31A...O5	0.84	1.95	2.707(5)	149.5
O5-H5A...O3	0.84	1.87	2.707(5)	178.4
O1-H11A...O2	0.84	2.02	2.745(5)	143.5
O2-H25A...O1	0.84	1.91	2.745(5)	173.6
O3-H31A...O5	0.84	1.95	2.707(5)	149.5
O5-H5A...O3	0.84	1.87	2.707(5)	178.4
O1-H11A...O2	0.84	2.02	2.745(5)	143.5
O2-H25A...O1	0.84	1.91	2.745(5)	173.6
O3-H31A...O5	0.84	1.95	2.707(5)	149.5
O5-H5A...O3	0.84	1.87	2.707(5)	178.4
O1-H11A...O2	0.84	2.02	2.745(5)	143.5
O2-H25A...O1	0.84	1.91	2.745(5)	173.6
O3-H31A...O5	0.84	1.95	2.707(5)	149.5
O5-H5A...O3	0.84	1.87	2.707(5)	178.4
O1-H11A...O2	0.84	2.02	2.745(5)	143.5
O2-H25A...O1	0.84	1.91	2.745(5)	173.6
O3-H31A...O5	0.84	1.95	2.707(5)	149.5
O5-H5A...O3	0.84	1.87	2.707(5)	178.4
O1-H11A...O2	0.84	2.02	2.745(5)	143.5
O2-H25A...O1	0.84	1.91	2.745(5)	173.6
O3-H31A...O5	0.84	1.95	2.707(5)	149.5
O5-H5A...O3	0.84	1.87	2.707(5)	178.4
O1-H11A...O2	0.84	2.02	2.745(5)	143.5
O2-H25A...O1	0.84	1.91	2.745(5)	173.6
O3-H31A...O5	0.84	1.95	2.707(5)	149.5
O5-H5A...O3	0.84	1.87	2.707(5)	178.4
O1-H11A...O2	0.84	2.02	2.745(5)	143.5
O2-H25A...O1	0.84	1.91	2.745(5)	173.6
O3-H31A...O5	0.84	1.95	2.707(5)	149.5
O5-H5A...O3	0.84	1.87	2.707(5)	178.4
O1-H11A...O2	0.84	2.02	2.745(5)	143.5
O2-H25A...O1	0.84	1.91	2.745(5)	173.6
O3-H31A...O5	0.84	1.95	2.707(5)	149.5
O5-H5A...O3	0.84	1.87	2.707(5)	178.4
O1-H11A...O2	0.84	2.02	2.745(5)	143.5
O2-H25A...O1	0.84	1.91	2.745(5)	173.6
O3-H31A...O5	0.84	1.95	2.707(5)	149.5
O5-H5A...O3	0.84	1.87	2.707(5)	178.4
O1-H11A...O2	0.84	2.02	2.745(5)	143.5
O2-H25A...O1	0.84	1.91	2.745(5)	173.6
O3-H31A...O5	0.84	1.95	2.707(5)	149.5
O5-H5A...O3	0.84	1.87	2.707(5)	178.4
